# Acidic Oxidative Depolymerization Towards Functionalized Low-Molecular-Weight Lignin and High-Value-Added Aliphatic Monomers: Operating Conditions, Scale-Up, and Crosslinking

**DOI:** 10.3390/ijms26104872

**Published:** 2025-05-19

**Authors:** Marta C. Lourenço, Talita Nascimento, Pedro José Sanches Filho, Ana C. Marques, Marta Ramos-Andrés

**Affiliations:** 1 Centro de Recursos Naturais e Ambiente (CERENA), Instituto Superior Técnico, Universidade de Lisboa, Av. Rovisco Pais, 1049-001 Lisbon, Portugal; martaclourenco@tecnico.ulisboa.pt (M.C.L.); talita.nascimento@tecnico.ulisboa.pt (T.N.); 2 Centro de Química Estrutural (CEQ), Instituto Superior Técnico, Universidade de Lisboa, Av. Rovisco Pais, 1049-001 Lisbon, Portugal; pedro.filho@tecnico.ulisboa.pt; 3 CERENA, Department of Chemical Engineering, Instituto Superior Técnico, Universidade de Lisboa, Av. Rovisco Pais, 1049-001 Lisbon, Portugal

**Keywords:** Lignoboost, hydrogen peroxide, dicarboxylic acids, functionalization, Kraft lignin, acidic oxidative depolymerization

## Abstract

Lignin, a complex aromatic biopolymer abundant as waste in biorefineries and the pulp and paper industry, holds significant potential for valorization. This study presents the oxidative depolymerization of Lignoboost lignin (LB) using H_2_O_2_ under mild, solvent- and catalyst-free, inherently acidic conditions at 50–70 °C. The process aimed to produce functionalized low-molecular-weight oligomers, retaining aromaticity, and aliphatic dicarboxylic acids, rather than complete monomerization. The depolymerized LB was rich in aromatic dimers-trimers (68.6 wt.%) with high functionalization (2.75 mmol/g OHphen, 3.58 mmol/g OHcarb, 19.5 wt.% of H in -CH=CH-), and aliphatic dicarboxylic acids (53.4 wt.% of monomers). Acidic conditions provided higher depolymerization and functionalization than alkaline, alongside simplified product recovery. The process was also successfully applied to Kraft lignin, demonstrating versatility and robustness even with higher polymeric content feedstocks. The optimized conditions were scaled up (×25), improving efficiency and yielding Mw 464 g/mol and Đ 1.3. As proof of concept, the scaled-up product underwent radical crosslinking, resulting in a new biopolymer with higher thermal stability than LB (54.2 wt.% residual mass at 600 °C versus 36.1 wt.%). This green, scalable process enhances lignin valorization by producing functionalized low-molecular-weight lignin oligomers and dicarboxylic acids that can be used independently or together to form crosslinked networks.

## 1. Introduction

Lignin, the most abundant aromatic biopolymer in lignocellulosic biomass, is a significant component of the waste stream in biorefineries, particularly in the paper and bioethanol industries. Approximately 50 million tons of lignin are produced annually as a byproduct of the pulp and paper industry, with over 95% currently being burned for energy recovery [1]. Despite this underutilization, lignin holds great potential for valorization due to its high carbon content and aromatic structure, making it an attractive candidate for various applications, including biofuels, chemicals, and materials. The global lignin market was valued at approximately USD 995 million in 2023 and is projected to reach USD 1.33 billion by 2029, reflecting a growing interest in lignin-based products [2].

Kraft lignin (KL), derived from the Kraft pulping process, is one of the most studied types of lignin. However, it is characterized by a high molecular weight and inherent heterogeneity, making it poorly reactive and difficult to dissolve [3]. Moreover, KL typically contains higher levels of impurities, such as carbohydrates and inorganic compounds, which affect its reactivity [4]. As a result, KL often requires further processing to enhance its usability for a range of applications. In contrast, the Lignoboost process, a novel technique for lignin recovery, yields a lignin of higher purity and quality. The process involves the precipitation of lignin from black liquor using CO_2_, followed by intensive washing at pH 2 to remove residual impurities [5]. This approach results in a lignin with a more protonated character, which improves its reactivity and makes it more suitable for functionalization [6]. Nevertheless, improving purity alone is not sufficient; tailored depolymerization and functionalization are needed to modify lignin’s chemical structure and properties for advanced applications.

The main challenge with lignin lies in its complex, heterogeneous structure, which leads to low reactivity and poor solubility, even when a major fraction consists of oligomers. Depolymerization of lignin is widely studied not only to reduce its molecular weight and degree of polymerization (DP), but also to enhance its chemical functionality—such as the introduction of phenolic hydroxyl (OHphen), carboxylic, and unsaturated groups—and to improve processability. Reducing molecular weight offers several benefits, including reduced steric hindrance, decreased heterogeneity, increased reactivity, and improved solubility. The OHphen groups present in lignin are the most abundant and reactive functional groups [7]. Preserving these groups during depolymerization is crucial for maintaining lignin’s reactivity. Oxidative depolymerization is an effective method to achieve this goal, as it can partially preserve OHphen groups while generating new functional groups, particularly carboxylic hydroxyl groups (OHcarb), which are highly reactive and have potential applications in processes like crosslinking [8]. In addition, oxidative depolymerization leads to the formation of alkene groups (aliphatic double bonds), which, when adjacent to C-C bonds, form vinyl groups that are highly reactive in radical polymerization reactions [9]. In addition to low-molecular-weight functionalized aromatics, one of the most common derivatives obtained from oxidative depolymerization are dicarboxylic acids, which can be generated through the cleavage of the aromatic ring [10]. These molecules have wide-ranging industrial applications, particularly in the synthesis of polyesters or biopolyesters.

Various methods for lignin oxidative depolymerization have been extensively studied, with hydrogen peroxide (H_2_O_2_) emerging as one of the most promising oxidizing agents due to its accessibility, low cost, and environmentally friendly byproducts (water and oxygen). H_2_O_2_ has been shown to depolymerize lignin under both acidic and alkaline conditions under different mechanisms [11]. One of the key differences lies in the solubility of lignin; while it is insoluble in acidic environments, it becomes soluble in alkaline conditions.

Previous studies have explored the oxidative depolymerization of lignin with H_2_O_2_ for functionalization while preserving the oligomeric structure. Ahmad et al. (2020) successfully depolymerized KL under inherent acidic conditions and ambient temperature, achieving functionalization and molecular weight reduction, leading to functionalized oligomers [12]. Junghans et al. (2020) reported alkaline oxidative depolymerization with diluted H_2_O_2_ at moderate temperatures (30–90 °C), achieving an 82% molecular weight reduction through side chains cleavage while preserving β-O-4 linkages. The oligomers were functionalized with increased COOH content (2.66 mmol/g) [13]. Ruwoldt et al. (2025) performed alkaline oxidation with H_2_O_2_ to convert OHphen groups into OHcarb, but this process did not lead to depolymerization and instead lignin’s molecular weight increase, although enhancing its solubility for emulsifier applications [14].

The advantages of acidic over alkaline depolymerization prompted Kim et al. (2020) to study the depolymerization of lignin by combining acetic acid with H_2_O_2_ [15]. This combination allowed for aromatic ring cleavage and COOH incorporation, producing polycarboxylates with applications as plasticizers. Li et al. (2020) employed H_2_O_2_ with formic acid and a mineral acid catalyst to cleavage C-C and β-O-4 linkages at ambient temperature, generating aromatic monomers (phenoxy-methyl benzoate, benzoic acid, and phenol) as the main products (90%) [16]. More recently, Andriani and Lawoko (2024) combined acetic acid and H_2_O_2_, finding that ring cleavage led to the formation of muconic acid- and ester-end groups and at the same time kept the macromolecular structure of the lignin intact, but in an oxidized form, resulting in the recovery of multifunctionalized oligomers with COOH groups [3]. These oligomers were less dispersed than the original lignin and exhibited more hydrophilic characteristics.

Regarding the generation of low-molecular-weight aliphatic derivatives, catalysts have often been employed to enhance production. Vega-Aguilar et al. (2021) studied the production of C4 dicarboxylic acids by oxidizing various lignins with H_2_O_2_ and titanium silicalite-1 as a catalyst [17]. This enabled the production of succinic acid as the major compound. Sun et al. (2023) studied the electrochemical conversion of lignin into short-chain carboxylic acids using H_2_O_2_ with titanium silicate-1 as a catalyst, generating H_2_O_2_ in situ [18]. Bi et al. (2018) also studied lignin degradation into dicarboxylic acids using CuFeS_2_ nanoparticles as a catalyst in combination with H_2_O_2_, with oxalic acid being the major product (30% selectivity) [19].

To the best of our knowledge, no other research group has fully described the acidic oxidative depolymerization of lignin under the specific conditions of high H_2_O_2_ concentration, high lignin concentration, and without the addition of external acids, co-solvents, or catalysts. This process achieves high efficiency in obtaining depolymerized/functionalized lignin, along with dicarboxylic acids. The acidic environment is created through the inherent protonated character of Lignoboost lignin (LB), which is suspended at a high concentration in water. This simplifies product recovery via simple drying and enhances scalability. This recovery method ensures that all derivatives formed during depolymerization are preserved without loss, a common issue in processes that involve precipitation. The present study investigates the effects of temperature and time on LB depolymerization, as well as the impact of operating under inherent acidic versus alkaline conditions, and the influence of stirring in acidic conditions. The optimized process was applied to both LB and KL, identifying the necessary adjustment for adapting the process to less pure lignins. Finally, the process was successfully scaled up by a factor of 25, incorporating improvements that resulted in a higher degree of depolymerization. As proof of concept, the depolymerized products were crosslinked by free-radical reactions, leading to a derivative with significantly enhanced thermal stability, making it a promising candidate for future studies and a wide range of applications.

## 2. Results and Discussion

### 2.1. Acidic Oxidative Depolymerization Under Different Temperatures

#### 2.1.1. Molecular Weight Distribution During Acidic Oxidative Depolymerization

**Figure 1:**
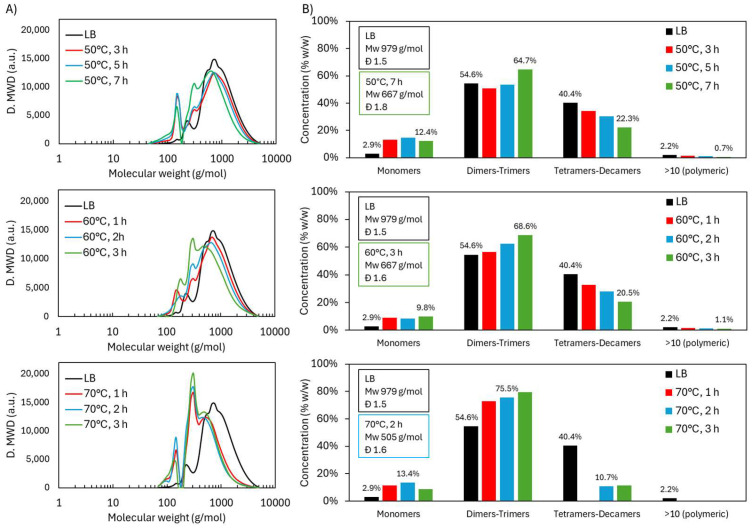
Molecular weight distribution of Lignoboost lignin during acidic oxidative depolymerization under different temperatures: (**A**) GPC chromatograms and (**B**) distribution in molecular groups.

The molecular weight distribution of the acidic oxidative depolymerization at the three operational temperatures (Figure 1A) showed the emergence of two distinct peaks corresponding to lower molecular weights. The first peak, located around 150 g/mol, was associated with the monomers’ presence and initially increased in the samples, gradually decreasing thereafter. Typical lignin monomers with molecular weights near 150 g/mol include vanillin (152 g/mol), vanillic acid (168 g/mol), coumaric acid (164.05 g/mol), malic acid (134.10 g/mol) and succinic acid (118.10 g/mol), among others. The second peak, around 300 g/mol, likely corresponds to dimers or trimers, depending on the level of functionalization of the molecules. This peak consistently increased over time. When comparing the temperatures, it can be observed that the distribution obtained at 50 °C after 7 h was very similar to that achieved at 60 °C after just 3 h. In contrast, at 70 °C, only 2 h were required to reach a distribution that appeared stable. This distribution displayed the highest degree of depolymerization, with a greater increase in the monomer and dimers-trimers peaks and a more significant reduction in the oligomeric region.

The proportion of different molecular groups (Figure 1B) was estimated following the method described in Section 3.4. The results show that the number of monomers increased slightly, reaching a maximum of 15.8 wt.% after 50 °C, 5 h. In the later samples, a slight decrease was observed, which could be attributed to possible repolymerization processes. On the other hand, the dimers-trimers fraction increased over time, reaching 64.7 wt.% (50 °C, 7 h), 68.6 wt.% (60 °C, 3 h), and 75.5 wt.% (70 °C, 2 h). The group of tetramers–decamers showed a marked decrease, which was more pronounced at higher temperatures, decreasing from 40.4 wt.% in the original lignin to a minimum of 22.3 wt.% (50 °C, 7 h), 20.5 wt.% (60 °C, 3 h), and 10.7 wt.% (70 °C, 2 h). The polymeric fraction was consistently minor, with percentages below 1.1 wt.%.

These results demonstrate an effective depolymerization process toward functionalized low-molecular-weight oligomers, where the monomers are not the majority compounds. However, dimers-trimers molecules reach a very high proportion, thereby increasing the homogeneity of the lignin-based material and reducing its steric hindrance. Furthermore, this process successfully led to the presence of more new functional groups, as will be discussed in the next section.

#### 2.1.2. Structural Characterization (ATR-FTIR, ^1^H NMR, ^31^P NMR, EA, TGA, GC-FID/(TOF-MS))

To better understand the evolution of functional groups during acidic oxidative depolymerization of lignin, structural characterization was performed using multiple complementary techniques.

**Figure 2:**
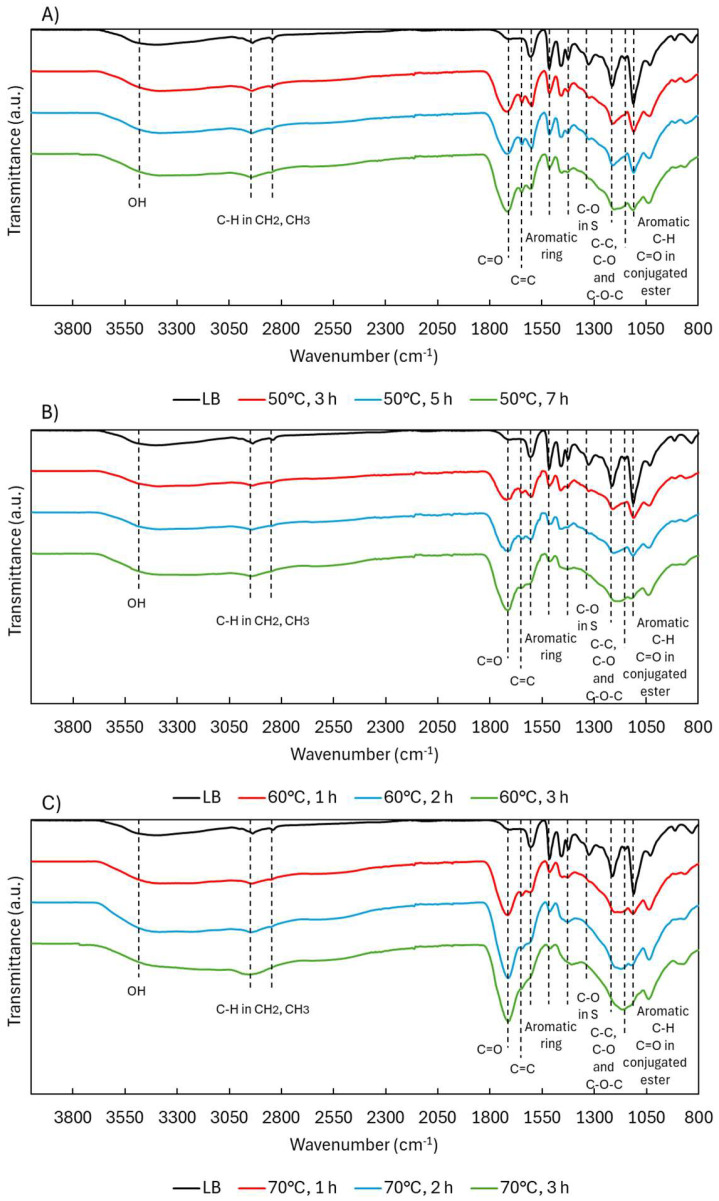
ATR-FTIR spectra of Lignoboost lignin during acidic oxidative depolymerization under different temperatures: (**A**) 50 °C, (**B**) 60 °C, and (**C**) 70 °C.

ATR-FTIR analysis (Figure 2) showed that OH bands (3600–3000 cm^−1^) [20] remained present in all depolymerized samples, although shifts in OH types were later confirmed through NMR. Peaks corresponding to aliphatic C–H stretching in CH_2_ and CH_3_ groups (2938, 2842 cm^−1^) [21] were consistently observed, with slightly broader signals at 70 °C, possibly reflecting increased surrounding functional group diversity.

**Figure 3:**
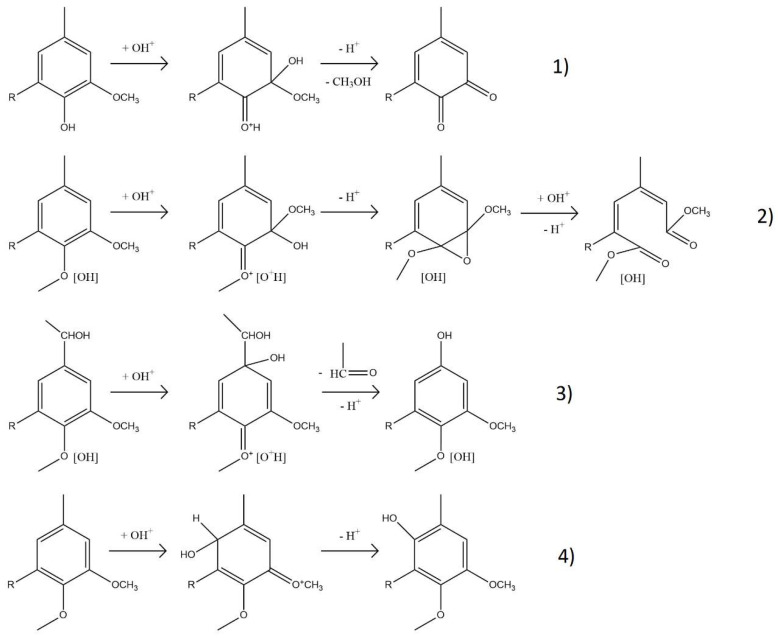
Proposed mechanisms occurring during lignin oxidative depolymerization in acidic H_2_O_2_ media: (**1**) demethoxylation, (**2**) ring cleavage, (**3**) displacement of side chains, and (**4**) ring hydroxylation. Adapted from Gierer (1986) [23].

The most prominent change occurred at 1714 cm^−1^, where the carbonyl (C=O) band [12] increased in intensity with both temperature and time. This is likely due to the formation of carboxylic acids, ketones, aldehydes, and esters, generated through oxidative cleavage of ether linkages and side chains. A new band at 1642 cm^−1^, attributed to aliphatic C=C bonds [22], appeared under all conditions but was most intense at lower temperatures. These alkenes likely form through ring cleavage or the creation of quinonoid structures, as illustrated in Figure 3, and are relevant markers of lignin reactivity. The illustrated mechanisms represent oxidative modification of lignin promoted by hydroxyl radical generated under acidic H_2_O_2_ conditions, rather than classical hydrolytic cleavage via Hibbert ketone formation.

The three primary aromatic bands at 1604, 1515, and 1424 cm^−1^ [12] remained largely unchanged for the first two peaks, indicating the aromatic backbone was mostly preserved despite depolymerization. The 1424 cm^−1^ band, however—associated with in-plane ring deformation—decreased notably and disappeared at higher temperatures, possibly due to substitution pattern changes or side-chain removal.

The syringyl (S) C–O band at 1323 cm^−1^ [12] also decreased, suggesting demethoxylation via OCH_3_ loss. The region around 1214 cm^−1^ (C–C, C–O, and C–O–C) [5,21] remained present but began overlapping with the 1145 cm^−1^ band, which increased with temperature and is assigned to esters conjugated with aromatics or unsaturated systems [24]. This suggests ester formation via ring cleavage or acid-catalyzed esterification between COOH and OH groups. Supporting this, the 1132 cm^−1^ peak (aromatic C–H in-plane deformation) [25] gradually decreased, though never fully disappeared, pointing to progressive side-chain removal.

**Figure 4:**
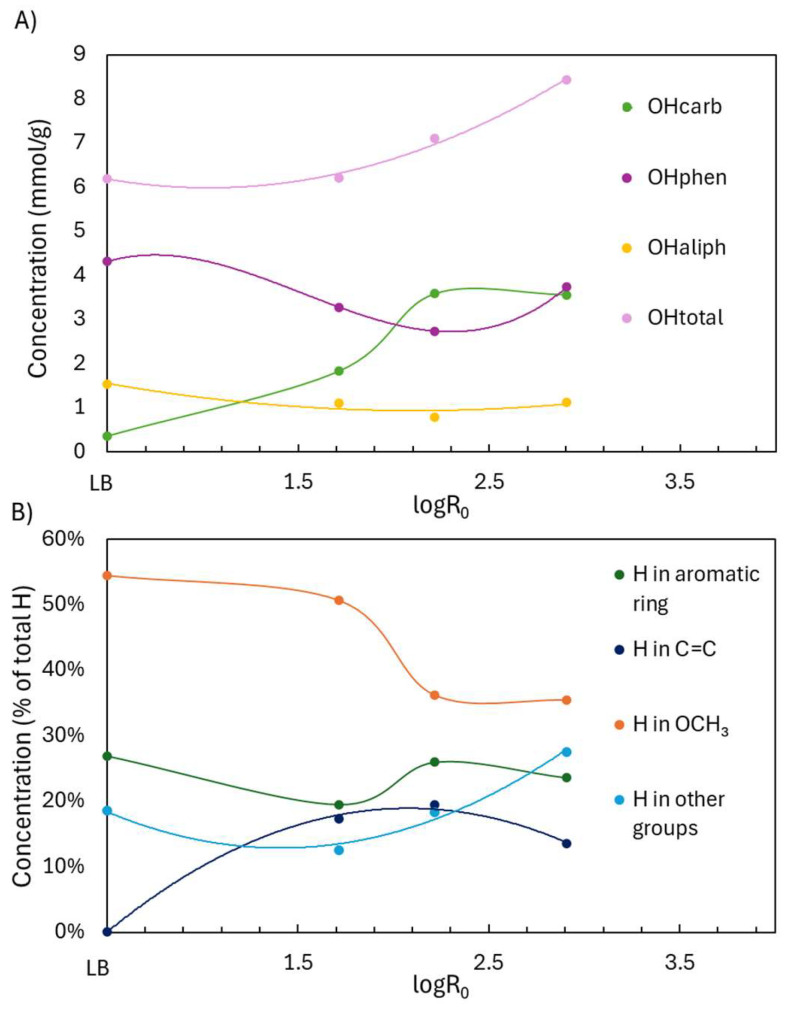
Functional group analysis of Lignoboost lignin after acidic oxidative depolymerization under different severity factors: (**A**) hydroxyl group content from ^31^P NMR and (**B**) hydrogen distribution from ^1^H NMR.

^31^P NMR analyses (Figure 4A) provided a clearer view of how specific OH groups evolved with depolymerization severity, expressed as logR_0_. For logR_0_ < 2.2, ^31^P NMR showed a decline in both OHphen and aliphatic hydroxyl (OHaliph) groups, alongside a substantial increase in OHcarb, suggesting oxidation of OH groups into acids or quinones. As severity increased beyond logR_0_ = 2.2, OHphen slightly recovered, while OHcarb plateaued, indicating that further oxidation was limited under these conditions. At 60 °C for 3 h (logR_0_ = 2.2), the OH content shifted from 0.35 to 3.58 mmol/g (OHcarb), 4.31 to 2.74 mmol/g (OHphen), and 1.53 to 0.78 mmol/g (OHaliph), with OHtotal increasing from 6.20 to 7.10 mmol/g. These results demonstrate an effective functionalization process (COOH, C=C) while largely preserving the OHphen groups. ^31^P NMR spectra can be found in Supplementary Material (Figures S11–S14).

^1^H NMR trends mirrored these changes (Figure 4B). Below logR_0_ = 2.2, OCH_3_ protons declined due to demethoxylation, while C=C protons increased as side-chain and ring cleavages produced unsaturated compounds, including unsaturated esters and carboxylic/dicarboxylic acids. The mechanisms of demethylation and C=C formation are illustrated in Figure 3. Aromatic H content remained relatively stable, as the cleavage of rings was offset by the liberation of aromatic protons from broken side chains. Above logR_0_ = 2.2, OCH_3_ proton content stabilized, and both aromatic and C=C protons slightly decreased—likely due to saturation and transformation into aliphatic groups. At logR_0_ = 2.2, H in OCH_3_ dropped from 54.5% to 36.2%, H in C=C rose from 0.1% to 19.5%, and aromatic-H fell modestly from 26.9% to 25.9%. Thus, the aromaticity was preserved, OCH_3_ groups decreased, and C=C groups were gained. ^1^H NMR spectra can be found in Supplementary Material (Figures S1–S4).

Elemental analysis (Table 1) further confirmed oxidative incorporation. C content declined steadily with severity, from 57.99% (LB) to 50.73%, 48.56%, and 46.48% at increasing temperatures. This was accompanied by a rise in O content from 32.86% to 43.46%, due to functionalization with OH, COOH, and C=O groups, as well as due to possible C reduction through gas-phase losses (CO_2_, CO). H content showed minor variation. Based on these data and OCH_3_ quantification, the empirical formula of the phenyl propane unit (PPU) for each depolymerized lignin was calculated. Despite demethoxylation reducing molar mass, the increase in oxygen content raised the PPU mass from 235.69 g/mol (LB) to 262.24, 255.60, and 271.28 g/mol for logR_0_ values of 1.7, 2.2, and 2.9, respectively.

**Figure 5:**
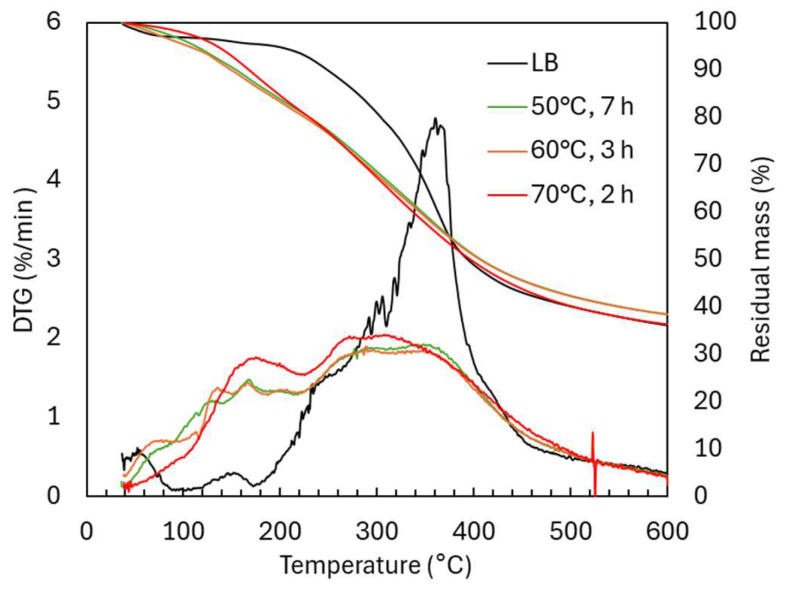
TG and DTG curves in Lignoboost lignin after acidic oxidative depolymerization under different severity factors.

Thermogravimetric analysis (TGA) and derivative thermogravimetry (DTG) in Figure 5 revealed clear trends correlating with depolymerization degree. The 150–230 °C region, associated with volatile monomers, showed increasing degradation: 10.6%, 10.5%, and 12.9 wt.% at 50 °C, 60 °C, and 70 °C, respectively. These closely matched the GPC-estimated monomer yields of 12.4%, 9.8%, and 13.4%. The 230–310 °C region, likely involving oligomeric species and functional group breakdown, showed a smaller increase in degradation with temperature (13.3%, 13.3%, 14.7%). In the 310–400 °C region, linked to high-molecular-weight oligomers, degradation remained similar across samples (~15.2–15.8%), though DTG peaks flattened with increasing severity. The residual mass at 600 °C remained comparable to LB (36.1%), with minor variation across depolymerized samples (36.3–38.4%).

Some of the monomers formed during depolymerization at the three temperatures were identified by GC-FID/(TOF-MS) (Table 2), and the corresponding chromatograms and chemical structures are provided in Supplementary Material (Figures S21–S24 and Figures S25–S31, respectively). The results from ^31^P NMR showed a clear prevalence of OHphen over OHaliph, which suggests a greater presence of aromatic rather than aliphatic compounds. However, the identification of monomers by GC-FID/(TOF-MS) showed a predominance of aliphatic compounds, which likely arise from the multiple transformations occurring, including the cleavage of side chains and the cleavage of aromatic rings (Figure 3). The absence of dimers and trimers in the GC-FID/(TOF-MS) analysis could be attributed to a lack of solubility and/or volatility even after derivatization.

Among the monomers identified, there was a strong formation of low-molecular-weight dicarboxylic acids: 50.28, 53.40, and 55.21 wt.% for logR_0_ values of 1.7, 2.2, and 2.9. Oxalic and propanoic acids, containing 2 and 3 C atoms, were most abundant, suggesting oxidative side-chain cleavage and possible ring cleavage followed by decarboxylation of the resulting muconic acids. Notably, no muconic acids (i.e., six-carbon dicarboxylic acids) were detected. Considering the estimated proportion of monomers from GPC analysis, the content of dicarboxylic acids in the solid depolymerized samples would be 6.23 wt.% (50 °C, 7 h, logR_0_ 1.7), 5.23 wt.% (60 °C, 3 h, logR_0_ 2.2), and 7.5 wt.% (70 °C, 2 h, logR_0_ 2.9).

Additionally, hydroxycarboxylic acids were abundant, likely originating from the breakdown of side chains attached to the aromatic rings [18]. Their presence was greater under more severe conditions, being 14.76, 14.93, and 16.93 wt.% for logR_0_ values of 1.7, 2.2, and 2.9, respectively. The major hydroxycarboxylic acids identified were glycolic acid, glyceric acid, and malic acid. As shown by the ATR-FTIR spectra, under more severe conditions, the formation of esters via esterification between COOH and OH, catalyzed by acidic conditions, was favored. The total content of esters increased from 1.65 wt.% (logR_0_ 1.7) to 11.28 wt.% (logR_0_ 2.9). The predominant esters were methyl 2-hydroxyethyl malonate and monomethyl succinate.

Regarding the total content of monomers identified, minor compounds included alcohols such as ethylene glycol (up to 2.05 wt.%) and lactones. Lactones are compounds formed by the cyclization of a COOH group with an OH group. Their presence increased as treatment severity increased, going from 0.54 wt.% (logR_0_ 1.7) to 2.39 wt.% (logR_0_ 2.9). Notably, the presence of monosaccharides was also observed, reaching 12.16 wt.% when the treatment conditions were milder (logR_0_ 1.7) and decreasing to 2.10 wt.% under more severe conditions (logR_0_ 2.9). This observation is unexpected, given that LB is of high purity and contains minimal carbohydrate content. One alternative hypothesis is that these sugar-like structures are formed via acidic hydrolysis and subsequent decarboxylation of lactones.

Finally, it is worth noting the content of aromatic compounds. The aromatics identified do not represent the entirety of those present but do provide an idea of which are the ones with lower molecular weight. The proportion of monomers identified as aromatics was 12.89, 12.57, and 2.73 wt.% for logR_0_ values of 1.7, 2.2, and 2.9, respectively. The decrease in aromatic monomers under more severe conditions seems to indicate that these units undergo further transformations, ranging from ring cleavage to some degree of repolymerization. The most abundant aromatic monomers that could be identified, after 60 °C, 3 h, were 4,4′-methylenedi-2,6-xylenol (up to 3.75 wt.%), syringic acid (up to 3.43 wt.%), acetyl syringic acid (up to 1.17 wt.%) and vanillic acid (up to 1.93 wt.%). Protocatechuic acid was also identified (up to 0.71 wt.%), which exhibits an interesting catechol functional group, with potential photoactivity and wide pharmacological activities.

These findings suggest that although aromatic ring opening does occur, supported by the detection of aliphatic C=C groups via ATR-FTIR and ^1^H NMR, a significant portion of the aliphatic monomers likely arises from the oxidative cleavage of side chains, which is often an unavoidable outcome in lignin depolymerization. Aliphatic dicarboxylic acids such as oxalic and propanedioic acids may also result from partial ring opening followed by decarboxylation. While these transformations may reduce the overall aromatic monomer yield, they do not necessarily compromise the value of the product stream. On the contrary, these oxygenated aliphatic compounds offer useful functionality for materials applications and may enhance reactivity.

### 2.2. Oxidative Depolymerization: Effect of Stirring and pH

#### 2.2.1. Molecular Weight Distribution

To examine the influence of operational variables, depolymerization experiments were compared at 60 °C for 3 h—selected as a reference condition due to the stabilization of functional group variations at this severity.

**Figure 6:**
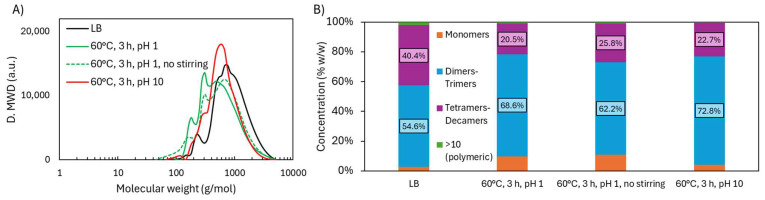
Effect of stirring and pH on the molecular weight distribution of Lignoboost lignin after oxidative depolymerization: (**A**) GPC chromatograms and (**B**) estimated distribution across molecular weight ranges.

Lignin remains suspended, not dissolved, in acidic oxidative depolymerization. Given its high concentration (300 mg/mL), adequate stirring is essential for effective mass transfer and depolymerization. Without stirring, aggregates form, limiting contact between H_2_O_2_ and the solid. This was reflected in molecular weight distribution (Figure 6), where the unstirred reaction produced fewer dimers–trimers (20.5 vs. 25.8 wt.%) and more tetramers–decamers (68.6 vs. 62.2 wt.%), indicating lower depolymerization efficiency.

H_2_O_2_ is also active in alkaline media, particularly between pH 10–12.5 [11]. Here, pH ~10 was used to balance oxidative power and minimize H_2_O_2_ decomposition. Under these conditions (60 °C, 3 h), monomer content was much lower (4.1 wt.%) compared to acidic treatment (9.8 wt.%). Dimers–trimers and tetramers–decamers were slightly higher (72.8 and 22.7 wt.%, respectively), suggesting less fragmentation. This reduced monomer recovery can be attributed to: (1) repolymerization, promoted by lignin’s solubility in alkaline media; (2) loss during precipitation, as monomers remained in solution upon pH adjustment to 2; and/or (3) incomplete depolymerization, due to greater structural stability under alkaline conditions. These phenomena—repolymerization, monomer loss during precipitation, and incomplete depolymerization—have been previously reported by other authors [13,14]. Monomer recovery from the liquid phase was considered, but the high salt content (60 wt.%) in that fraction made it unviable. Weight-average molecular weight (Mw) and dispersity (Đ) values were 667 g/mol and 1.6 under acidic conditions, compared to 705.6 g/mol and 1.4 under alkaline conditions.

Overall, depolymerization under acidic conditions is improved by stirring, which enhances contact between lignin particles and H_2_O_2_, whereas alkaline depolymerization results in lower monomer recovery, likely due to a combination of repolymerization, incomplete depolymerization, and product loss during precipitation.

#### 2.2.2. Structural Characterization (ATR-FTIR, ^1^H NMR, ^31^P NMR, EA, TGA)

This section compares the structural outcomes of oxidative depolymerization under acidic conditions without stirring and under alkaline conditions.

**Figure 7:**
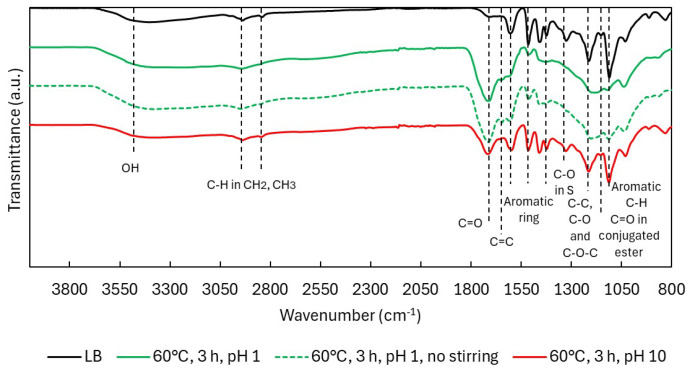
ATR-FTIR spectra in Lignoboost lignin after oxidative depolymerization: effect of stirring and pH.

The ATR-FTIR spectra (Figure 7) of the non-stirred acidic experiment closely resemble those obtained with stirring, with only minor differences. A slightly lower intensity of the carbonyl (C=O) band at 1714 cm^−1^ and a slightly higher intensity of the C–H in-plane aromatic signal at 1132 cm^−1^ suggest a lower formation of carboxylic acids and reduced demethoxylation.

Under alkaline conditions, the spectra resembled native LB, with minimal structural changes. The C=O oxidation peak appeared weakly, and no ester-related peak (1145 cm^−1^) was detected, implying no esterification. Peaks for OCH_3_-bound S units (1323 cm^−1^) and C–H aromatic vibrations (1132 cm^−1^) remained strong, indicating preserved aromatic substitution and minimal demethoxylation. The resulting lignin was structurally similar to the original LB, albeit with a slightly reduced Mw (708 vs. 979 g/mol).

**Figure 8:**
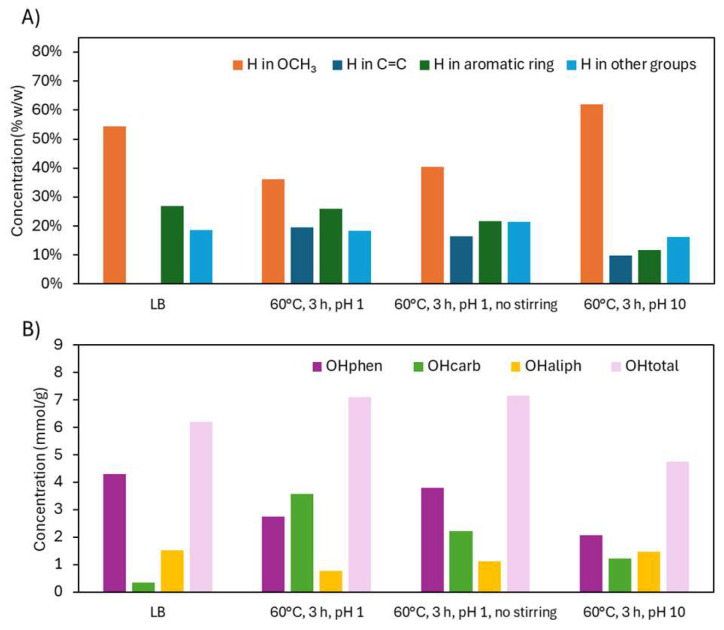
Functional groups analysis of Lignoboost lignin after oxidative depolymerization under different stirring and pH conditions: (**A**) hydrogen distribution from ^1^H NMR and (**B**) hydroxyl group content from ^31^P NMR.

The ^1^H NMR analysis (Figure 8A) reveals the distribution of protons across different functional groups. The corresponding spectra can be found in Supplementary Material (Figures S1, S3, S5 and S6). Under acidic conditions, demethoxylation occurred, reducing the OCH_3_ group attached to the aromatic ring. This led to a slight increase in aromatic protons, compensating for the loss of aromatic H through other reactions, keeping its overall level fairly constant. The C=C group increased, likely due to the cleavage of aromatic rings and side chains. When no stirring was applied, the process efficiency decreased, resulting in less demethoxylation, a smaller increase in C=C, and a more pronounced decrease in aromatic H compared to stirred conditions.

Under alkaline conditions, the proportion of OCH_3_ groups increased compared to acidic conditions. This could be due to the reduction of H from other groups, while OCH_3_ remained intact, as observed in the ATR-FTIR spectra. Previous studies have shown that demethoxylation occurs under acidic conditions but not under alkaline conditions [17]. The C=C group appeared in smaller amounts, and aromatic H decreased. The formation of quinonic structures may explain the increase in C=C and the decrease in aromatic protons, while the OCH_3_ groups remained intact. This process is promoted by the deprotonation of OHphen groups [11].

The analysis of OH groups (Figure 8B) shows that the increase in OHcarb, indicating oxidation, was most pronounced under acidic conditions with stirring, followed by lower oxidation under conditions without stirring and even less under alkaline conditions. Despite less oxidation without stirring, depolymerization still occurred, decreasing the molecular weight of LB (from 979 to 730 g/mol) and increasing monomer-dimer-trimer proportion (from 57.4 to 73.1 wt.%), while maintaining a relatively high OHphen concentration (from 4.31 to 3.81 mmol/g).

In alkaline conditions, OHcarb increased slightly, likely due to phenolate ions being less prone to ring cleavage. However, alkaline conditions caused a significant decrease in OHphen (from 4.31 to 2.07 mmol/g), more so than in acidic conditions. This is likely due to the deprotonation of OHphen groups, which increases their reactivity and promotes the formation of quinonic structures. The marked reduction in OHphen resulted in a decrease in OHtotal (from 6.20 to 4.76 mmol/g), which was also reflected in the ATR-FTIR spectrum. ^31^P NMR spectra can be found in Supplementary Material (Figures S11, S13, S15 and S16).

Elemental analysis (Table 3) shows that the non-stirred operation differs from the stirred one by a slightly lower increase in O content, resulting in less C loss and a smaller depletion of OCH_3_ groups. In contrast, alkaline depolymerization exhibits minimal C loss and O gain, indicating lower depolymerization and functionalization, but confirming a slight increase in OCH_3_ groups in the PPU. The molecular weight of PPU under acidic conditions was nearly identical with and without stirring (255.60 vs. 255.06 g/mol), despite differences in OCH_3_ content. However, PPU obtained under alkaline conditions, with more OCH_3_ groups but lower functionalization, had a lower molecular weight compared to the acidic depolymerization (246.26 vs. 255.60 g/mol).

**Figure 9:**
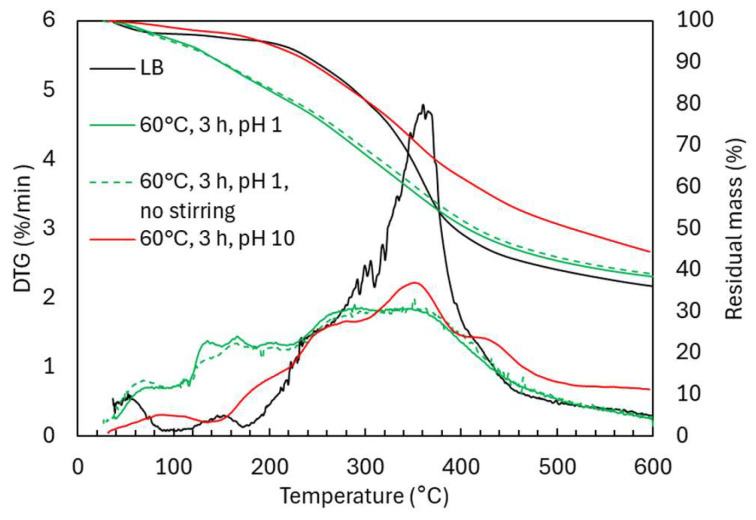
TG and DTG curves in Lignoboost lignin after acidic oxidative depolymerization: effect of stirring and pH.

The TG and DTG curves (Figure 9) for acidic oxidative depolymerization show a high similarity between experiments with and without stirring, with only a slightly higher proportion of oligomeric material (310–400 °C) in the non-stirred experiment.

In contrast, under alkaline conditions, the decomposition profile revealed distinct behaviors in different temperature regions. In the 150–230 °C region, which is linked to the evaporation of volatile monomers, alkaline-treated lignin exhibited a lower weight loss (5.6 wt.%) compared to acidic conditions (10.5 wt.%), consistent with GPC monomer estimates of 4.1 wt.% and 9.8 wt.% for alkaline and acidic conditions, respectively. In the 230–310 °C region, which involves the decomposition of functional groups and low-molecular-weight species, alkaline-treated lignin showed a slightly lower weight loss (12.3 wt.%) compared to acidic conditions (13.3 wt.%). In the 310–400 °C region, where maximum lignin degradation occurs (29.8 wt.% in LB), alkaline condition showed two degradation peaks: a major one at 360 °C and another at 428 °C. The second peak suggests the formation of thermally stable, crosslinked aromatic structures through repolymerization, consistent with previous studies [14]. This is supported by the higher degradation in the 400–600 °C region for alkaline-treated lignin (17.9 wt.% vs. 12.4 wt.% under acidic conditions and 12.7 wt.% in LB), and a higher residual mass at 600 °C (52 wt.% for alkaline-treated lignin compared to 38.4 wt.% under acidic conditions). These differences in thermal behavior arise from the distinct structural changes under acidic and alkaline conditions, with acidic depolymerization favoring side-chain cleavage and functional group introduction, and alkaline depolymerization retaining OCH_3_ groups and promoting thermal stability through repolymerization.

### 2.3. Oxidative Depolymerization of Kraft Lignin (KL) Versus Lignoboost Lignin (LB)

#### 2.3.1. Molecular Weight Distribution During Acidic Oxidative Depolymerization

**Figure 10:**
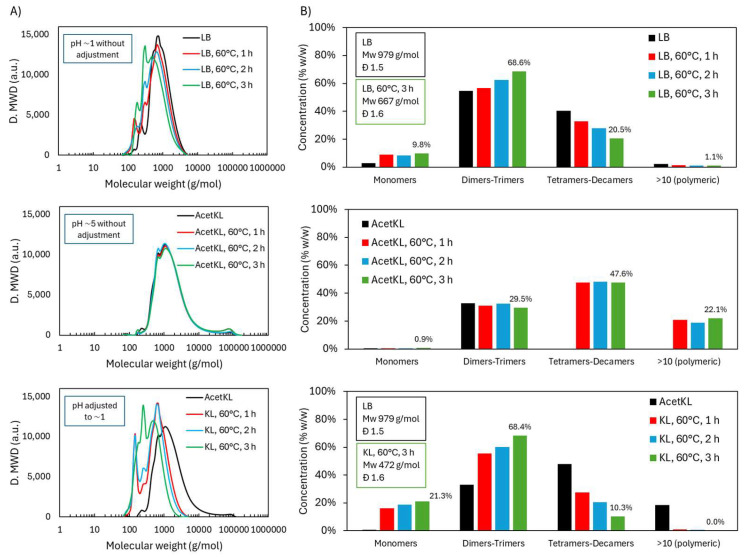
Molecular weight distribution during acidic oxidative depolymerization of Lignoboost lignin and Kraft lignin: (**A**) GPC chromatograms and (**B**) estimated distribution across molecular weight ranges.

To evaluate the versatility of the oxidative depolymerization method, the same acidic protocol applied to LB was used on another lignin type, KL. Both lignins originated from *Eucalyptus globulus*, but differ in purity and protonation levels due to their extraction process. Due to solubility limitations, KL was acetylated to enable its dissolution in THF for GPC analysis; this sample is referred to as “AcetKL” in Figure 10.

In replicating the LB-pH1-60 °C experiment with KL, the slurry pH reached ~5 due to KL’s lower protonation and higher salt content, which likely buffered the system. Although the medium was less acidic, the reaction was performed for comparison (KL-pH5-60 °C, Table 1). As shown in Figure 10, no significant depolymerization occurred; the molecular weight distribution remained unchanged over time. This result aligns with the fact that H_2_O_2_ is largely inactive near neutral pH due to low radical generation.

KL’s lower acidity posed a practical limitation, but using LB offers a key advantage: it introduces inherent acidity, avoiding external acid addition and minimizing salt content that is beneficial for scalability and product purity.

To assess KL under acidic conditions, the pH was adjusted to ~1 using 1 M H_2_SO_4_ (KL-pH1-60 °C). Under these conditions, depolymerization proceeded effectively. Initially, monomers peaked, followed by rising dimer and trimer fractions. The final distribution was 21.3 wt.% monomers, 68.4 wt.% dimers-trimers, and 10.3 wt.% tetramers–decamers (treating KL), compared to 9.8 wt.% monomers, 68.6 wt.% dimers-trimers, and 20.5 wt.% tetramers–decamers (treating LB). KL showed a more pronounced reduction in tetramers–decamers and a complete disappearance of the polymeric fraction, indicating higher depolymerization efficiency.

This enhanced efficiency in KL may be due to stable pH maintenance by strong acid (H_2_SO_4_), in contrast to LB’s self-generated acidity, which may fluctuate during the reaction. Additionally, catalytic impurities in KL may have promoted oxidative cleavage. Notably, depolymerized KL became THF-soluble without acetylation, suggesting altered solubility properties and reinforcing the structural transformation.

#### 2.3.2. Structural Characterization (ATR-FTIR, ^1^H NMR, ^31^P NMR, TGA, and GC-FID/(TOF-MS))

The structural differences between the depolymerized LB and KL were examined using multiple techniques.

**Figure 11:**
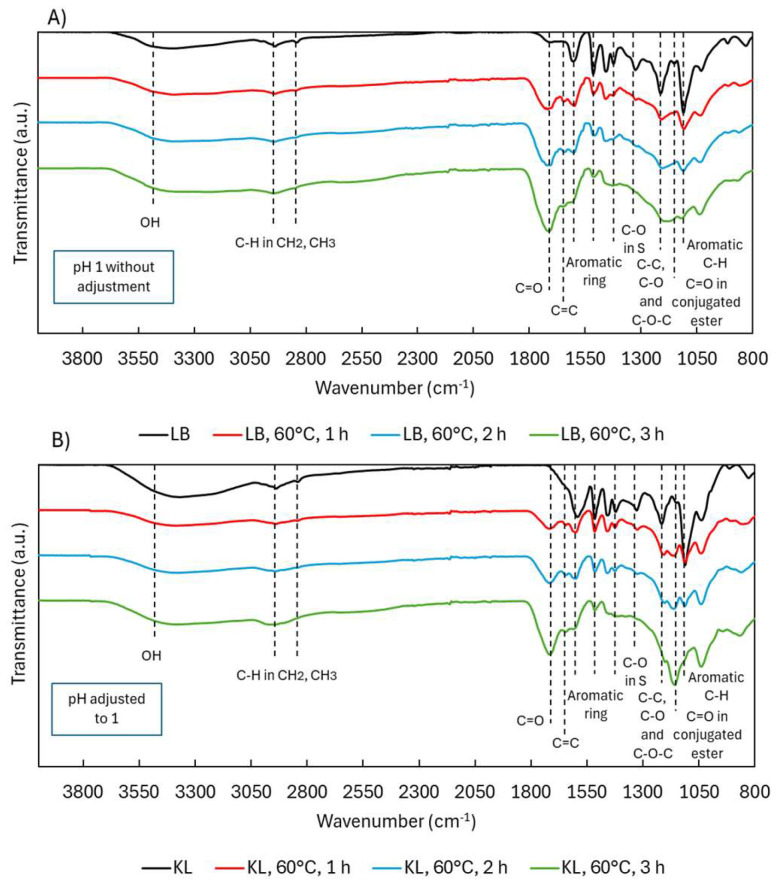
ATR-FTIR spectra during acidic oxidative depolymerization: (**A**) Lignoboost lignin and (**B**) Kraft lignin.

ATR-FTIR spectra (Figure 11) showed similar patterns in both lignins post-depolymerization, with KL displaying a notably stronger C=O band at 1145 cm^−1^ (conjugated esters). This suggests enhanced ester formation in KL, likely due to acid-catalyzed reactions facilitated by the stable pH maintained with H_2_SO_4_. These findings support the method’s applicability to less pure lignins like KL, not just high-purity LB.

**Figure 12:**
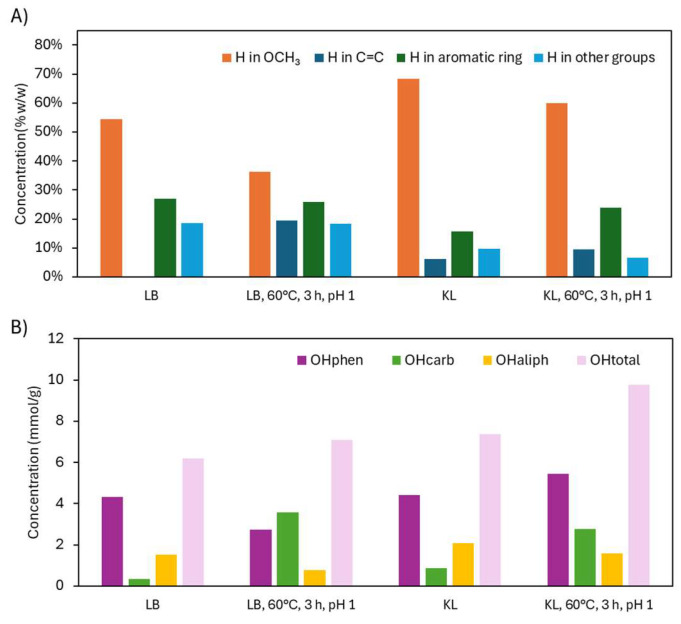
Functional group analysis of Lignoboost lignin and Kraft lignin after acidic oxidative depolymerization: (**A**) hydrogen distribution from ^1^H NMR and (**B**) hydroxyl group content from ^31^P NMR.

^1^H NMR analysis (Figure 12A) revealed that native KL contained more OCH_3_ groups than in LB, resulting in fewer aromatic protons due to increased substitution. After depolymerization, OCH_3_ signals decreased—consistent with demethoxylation—while aromatic proton signals slightly increased in KL. C=C signals also increased but to a lesser extent than in LB, possibly because KL’s OHphen groups were more stable under the stronger acid conditions, limiting quinone formation. ^1^H NMR spectra can be found in Supplementary Material (Figures S1, S3, S7 and S8).

^31^P NMR (Figure 12B) confirmed these trends. In KL, OHphen groups increased significantly (4.42 to 5.44 mmol/g), likely due to acid-stable conditions and potential aromatic hydroxylation. OHcarb groups also increased (0.86 to 2.76 mmol/g), while OHaliph groups decreased slightly (2.09 to 1.58 mmol/g), mirroring LB results. The OHtotal content rose from 7.37 to 9.78 mmol/g, indicating a more reactive and less sterically hindered lignin. This aligns with the sharp drop in molecular weight from 2981 to 472 g/mol. ^31^P NMR spectra can be found in Supplementary Material (Figures S11, S13, S17 and S18).

**Figure 13:**
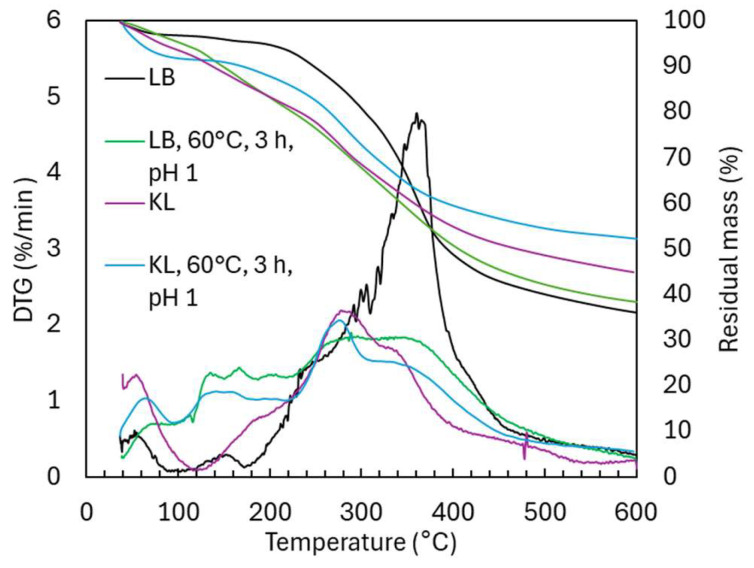
TG and DTG curves after acidic oxidative depolymerization: Lignoboost lignin versus Kraft lignin.

TGA/DTG analysis (Figure 13) showed that LB degraded primarily at 310–400 °C, while KL degraded at lower temperatures (230–310 °C), likely due to its higher OCH_3_ content, as demethoxylation typically occurs between 200 and 350 °C. KL also had more residual mass at 600 °C (52.3 wt.%) than LB (36.1 wt.%), likely reflecting higher impurity levels. After depolymerization, KL degraded at lower temperatures and had reduced residual mass (44.9 wt.%), consistent with lower molecular weight and possibly fewer impurities. In the 150–230 °C range (monomer degradation), both lignins showed minor weight loss, but depolymerized LB lost 10.5 wt.% and depolymerized KL 8.4 wt.%. Notably, no degradation peaks were observed between 400 and 600 °C, suggesting no crosslinked aromatic structures formed under acidic conditions.

GC-FID/(TOF-MS) analysis (Table 2) confirmed differences in the monomeric product profiles. Depolymerized KL contained more hydroxycarboxylic acids than LB (19.21 vs. 14.73 wt.%), with glycolic acid being predominant in both. This may reflect more efficient side-chain cleavage under stable acidic conditions. Conversely, KL had far fewer dicarboxylic acids (29.36 vs. 53.40 wt.%), especially oxalic acid, possibly due to reduced aromatic ring cleavage or stronger acid conditions favoring ester formation. In the depolymerization of KL, the content of OHphen increased, suggesting hydroxylation and less aromatic ring cleavage compared to LB, possibly due to the higher molecular weight of KL vs. LB (2981 vs. 979 g/mol).

Esterification under acidic conditions is supported by the higher ester content in KL (6.43 vs. 1.68 wt.%), with methyl 2-hydroxyethyl malonate as the main ester. KL also yielded more lactones (2.69 vs. 1.68 wt.%) and a notably higher monosaccharide content (29.9 vs. 10.87 wt.%), likely due to lignin–carbohydrate complexes co-precipitating during KL recovery from black liquor.

Aromatic monomers were present in low amounts in both cases, but even lower in KL (2.48 wt.%) than in LB (12.57 wt.%). However, NMR and FTIR data suggest that aromaticity remains high in oligomeric fractions (dimers, trimers). In KL, the predominant aromatic monomers were acetyl syringyl (1.26 wt.%) and vanillic acid (0.89 wt.%), while LB showed higher levels of 4,4′-methylenedi-2,6-xylenol (3.75 wt.%), syringic acid (3.43 wt.%), and vanillic acid (1.93 wt.%).

### 2.4. Scale-Up of Acidic Oxidative Depolymerization of Lignoboots Lignin (LB)

#### 2.4.1. Molecular Weight Distribution During Acidic Oxidative Depolymerization

**Figure 14:**
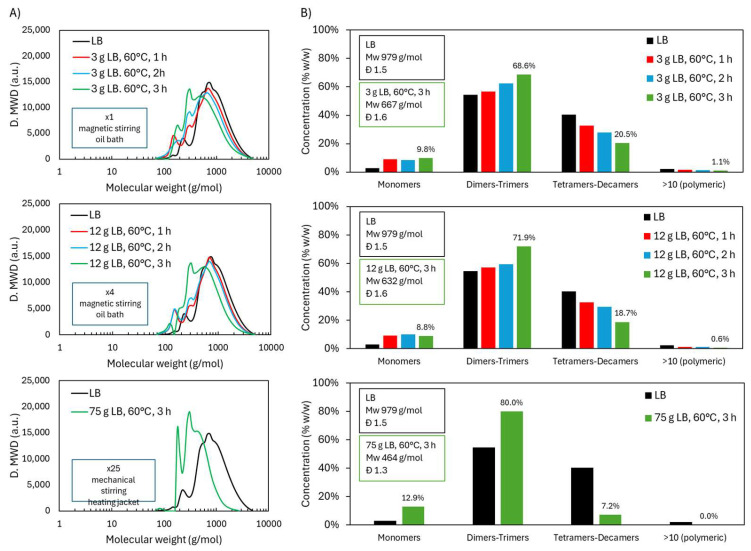
Molecular weight distribution of Lignoboost lignin during acidic oxidative depolymerization under different scales (×1, ×4, ×25): (**A**) GPC chromatograms and (**B**) estimated distribution across molecular weight ranges.

To enable broader applications of depolymerized lignin, the acidic oxidative depolymerization of LB was scaled up under the same reference conditions: inherent acidity, 60 °C, and a 3 h reaction time. The initial scale-up increased the reaction volume by a factor of 4, processing 12 g of LB in a reactor of the same type as the lab-scale setup, equipped with an oil bath and magnetic stirring. GPC results (Figure 14) confirmed that the molecular weight distribution remained virtually unchanged compared to the original scale. The product composition showed 8.8 wt.% monomers (vs. 9.8 wt.%), 71.9 wt.% dimers–trimers (vs. 68.6 wt.%), 18.7 wt.% tetramers–decamers (vs. 20.5 wt.%), and 0.6 wt.% polymeric fractions (vs. 1.1 wt.%). The Mw and Đ were also comparable: 667 g/mol and 1.6 at the smaller scale, versus 632 g/mol and 1.6 after the first scale-up. This similarity supports the consistency and repeatability of the methodology.

In the final stage, the process was scaled up 25-fold, treating 75 g of LB. This required a different reactor setup, using a water-jacketed vessel for more precise temperature control and a motorized mechanical stirrer to ensure uniform and vigorous mixing. These improvements significantly enhanced heat and mass transfer within the reaction medium, resulting in more effective dispersion of the lignin and better contact with the H_2_O_2_ oxidant. In contrast to small-scale setups with magnetic stirring, where there might be partial aggregation of solids that limits accessibility, the enhanced mixing at this scale likely prevented such effects, thereby facilitating a more complete depolymerization. The GPC chromatogram revealed a substantial reduction in higher molecular weight fractions. The polymeric region was completely eliminated, and the tetramers–decamers fraction dropped to 7.2 wt.% (vs. 20.5 wt.% at the original scale). Meanwhile, the dimers–trimers increased to 80.0 wt.% (vs. 68.6 wt.%) and the monomers to 12.9 wt.% (vs. 9.8 wt.%). This was reflected in a significantly lower Mw of 464 g/mol and a narrower dispersity (Đ = 1.3), compared to 667 g/mol and 1.6 at the original scale.

#### 2.4.2. Structural Characterization (ATR-FTIR, ^1^H NMR, ^31^P NMR, and TGA)

**Figure 15:**
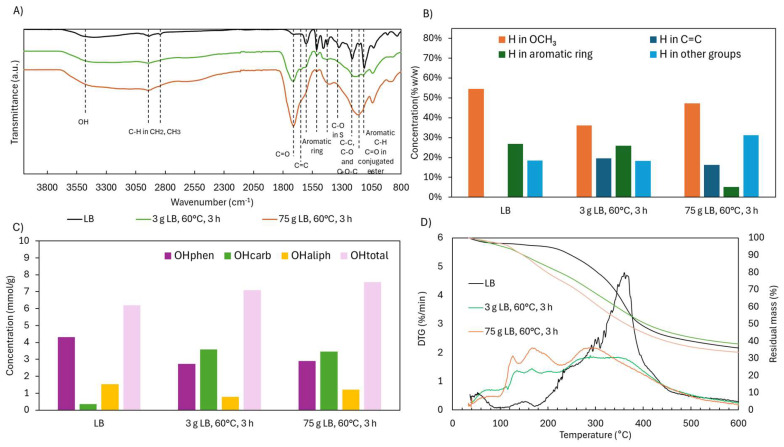
Functional group and thermal analysis of Lignoboost lignin after acidic oxidative depolymerization under different scales (×1, ×4, ×25): (**A**) ATR-FTIR spectra, (**B**) hydrogen distribution from ^1^H NMR, (**C**) hydroxyl group content from ^31^P NMR, and (**D**) TGA and DTG curves.

Given the enhanced depolymerization observed at the ×25 scale, structural differences between scales were assessed. The ATR-FTIR spectra (Figure 15A) were largely consistent between samples, though the scaled-up product showed a more intense band at 1145 cm^−1^, associated with conjugated esters. This suggests an increase in esterification reactions, likely driven by greater availability of COOH and OH groups under more homogeneous and efficiently stirred conditions. Improved mixing and temperature control likely played a key role in enhancing reaction uniformity and thus the extent of functionalization.

^1^H NMR analysis (Figure 15B) supported this interpretation (Supplementary Material, Figures S1, S3 and S9). The scaled-up reaction showed a notable reduction in aromatic H content (5.2% vs. 25.9% at the smaller scale), likely due to increased ring cleavage. Simultaneously, H associated with aliphatic groups increased, indicating enhanced breakdown of side chains. Interestingly, ^31^P NMR (Figure 15C) revealed little difference in the distribution of OH groups between the two scales, including the phenolic fraction (Supplementary Material, Figures S11, S13 and S19). This suggests that the drop in aromatic H content does not reflect a loss of aromatic structures per se, but rather a redistribution of H toward other functional groups, possibly with partial retention of OCH_3_ groups.

TG and DTG curves (Figure 15D) further corroborated the observed structural changes. In the 150–230 °C range—associated with the evaporation or degradation of volatile monomers—the larger-scale sample showed slightly greater mass loss (14.8 wt.% vs. 10.5 wt.%), consistent with its higher monomer content. Between 230 and 310 °C, corresponding to the degradation of OCH_3_ and COOH groups and some dimers/trimers, mass loss was again marginally higher at a larger scale (15.2 wt.% vs. 13.3 wt.%). In the primary degradation window of 310–400 °C, linked to aromatic breakdown, depolymerized samples showed a substantial reduction compared to native LB (29.8 wt.%). At the small and large scales, mass loss was 15.2 wt.% and 14.6 wt.%, respectively, suggesting comparable loss of aromaticity. Finally, in the 400–600 °C range, degradation remained relatively stable across all samples, decreasing slightly from 12.7 wt.% in native LB to 12.4 wt.% and 11.4 wt.% in the small- and large-scale products, respectively. Consistent with these thermal patterns and the increased degree of depolymerization, the residual mass at 600 °C was lower at the larger scale (33.6 wt.%) compared to the smaller-scale product (38.4 wt.%). This reduction reflects the formation of lower-molecular-weight, more oxidized, and thermally labile structures as the scale increases.

### 2.5. Reactivity of Depolymerized/Functionalized Lignoboost Lignin (LB) Through Radical Crosslinking

Previous studies have demonstrated that OHphen groups in lignin can be activated via redox initiator systems such as CaCl_2_/H_2_O_2_ under O_2_-free conditions and moderate temperatures [26,27]. However, to the best of our knowledge, this approach has not been applied to the self-crosslinking of depolymerized, highly functionalized lignin. Given that the depolymerized LB exhibits an enriched content of such functional groups, an activation test was carried out to assess their reactivity, specifically their ability to form crosslinked structures.

As proof of concept, the product from the ×25 scale-up experiment, hereafter referred to as Depolymerized Lignoboost Lignin (DLB), was subjected to radical-induced crosslinking using this redox system. During activation, the OHphen—along with other reactive functionalities introduced during depolymerization, such as C=C and OHcarb—participated in the formation of a partially crosslinked aromatic network. The resulting material, termed PolyActDLB, differs substantially from the original lignin and from DLB, as it represents a restructured and functionalized polymeric matrix.

**Figure 16:**
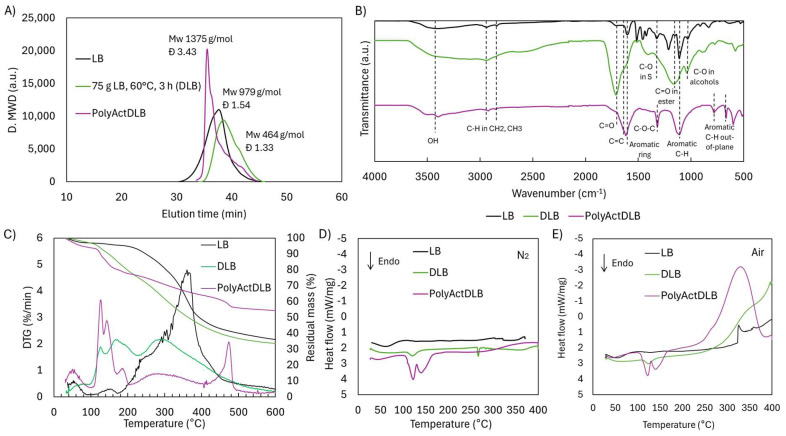
Molecular and structural characterization of lignin cross-linked matrix: (**A**) molecular weight distribution, (**B**) ATR-FTIR spectra, (**C**) TG and DTG curves, (**D**) DSC in N_2_ atmosphere, and (**E**) DSC in air atmosphere.

The comprehensive characterization of PolyActDLB confirmed the occurrence of crosslinking. The molecular weight distribution (Figure 16A) showed a pronounced increase, with Mw reaching 1375 g/mol, significantly higher than both DLB (464 g/mol) and the original LB prior to depolymerization (979 g/mol). The broadened dispersity (Đ = 3.43) further supports the formation of a highly branched polymer network, in contrast to the narrower distributions observed in LB and DLB (Đ = 1.33–1.54). Notably, PolyActDLB became insoluble in THF, unlike its precursors, necessitating a modified GPC protocol using DMF/LiCl as the solvent—additional evidence of successful crosslink formation and altered solubility behavior.

The ATR-FTIR spectra (Figure 16B) revealed clear structural changes in PolyActDLB compared to both LB and DLB. The broad OH band between 3600 and 3000 cm^−1^ was significantly reduced, likely due to the consumption of OH groups during the crosslinking reaction and the formation of new ether-type bonds. Interestingly, the prominent C=O peak at 1741 cm^−1^, evident in DLB, was absent in PolyActDLB. This could indicate that carboxylic and dicarboxylic acid groups either participated in bond formation or were lost during aqueous-phase filtration, as these low-molecular-weight compounds are water-soluble.

Despite these transformations, the aromatic character of the lignin backbone was largely preserved. The band at 1604 cm^−1^, associated with aromatic ring vibrations, remained prominent, as did the peak at 1132 cm^−1^ corresponding to C–H in-plane deformation in aromatic systems. A notable change was the appearance of a peak at 1318 cm^−1^, which is attributed to C–O–C stretching vibrations [28]. This peak may overlap with the signal typically assigned to the C–O bond in S units at 1323 cm^−1^; however, the C–O bond was present in the original LB and was almost completely reduced in DLB due to demethoxylation. Therefore, the 1318 cm^−1^ peak could be primarily attributed to C–O–C linkages, likely formed during crosslinking via reactions between activated OHphen groups and other reactive moieties. Two new bands emerged at 782 and 664 cm^−1^, assigned to C–H out-of-plane deformations in substituted aromatic rings [29]. These changes likely reflect alterations in ring substitution patterns or spatial orientation as a result of crosslinking.

Altogether, these spectroscopic features support the formation of a new bio-based aromatic polymer, of the polyether type. The proposed mechanism involves phenol activation followed by coupling, as illustrated below:

PPU–OHₚₕₑₙ → PPU–O* + H^+^ (activation with CaCl_2_/H_2_O_2_)

PPU–O* + PPU → PPU–O–PPU*

Thermal analysis by TGA and DTG (Figure 16C) showed that PolyActDLB exhibits remarkable thermal stability. Only a small fraction of the material (6.9 wt.%) decomposed below 320 °C, likely corresponding to unreacted DLB monomers. Unlike LB and DLB, which undergo major decomposition in the 230–400 °C range, PolyActDLB showed minimal degradation in this region. Instead, its primary degradation peak appeared between 400 and 500 °C, and even then, mass loss was only 8.1 wt.%. The residual mass at 600 °C was significantly higher—54.2 wt.%, compared to 36.1 wt.% for LB and 33.6 wt.% for DLB—demonstrating the formation of a stable, crosslinked aromatic network, which could be attractive for high-temperature applications such as carbon fiber precursors or flame-retardant materials.

DSC analysis under a N_2_ atmosphere (Figure 16D) further highlighted the differences between PolyActDLB and its precursors. While LB and DLB lacked distinctive thermal transitions—typical of amorphous, heterogeneous materials—PolyActDLB displayed two endothermic events between 100 and 150 °C. These may correspond to the release of residual unreacted monomers, degradation of weak crosslinks, or evaporation of activation byproducts. These thermal events were also observed under air (Figure 16E) and mirrored in the DTG curve.

Under oxidative conditions (Figure 16E), thermal responses diverged significantly. LB exhibited a mild exothermic slope beginning around 300–350 °C, likely due to oxidation of labile functional groups. DLB showed a steeper exothermic slope beginning at a lower temperature (250–300 °C), consistent with its higher functionality and increased C=O and OH group content. In PolyActDLB, the exothermic event began earlier, between 200 and 250 °C, peaking in the 350–400 °C range. This suggests that the newly formed C–O linkages in the crosslinked matrix are more susceptible to oxidative cleavage, unlike the more resilient aromatic structures in LB and DLB.

The ^1^H analyses showed interesting results (Supplementary Material Figure S10). On the one hand, the proton distribution in the different functional groups of PolyActDLB revealed preservation of H in aromatic rings (5.0 vs. 5.2 wt.%, 6.00–9.00 ppm), indicating that the aromatic rings were largely retained. In contrast, the OCH_3_ proton content decreased significantly (20.2 vs. 47.3 wt.%, 3.46–4.00 ppm). Since OCH_3_ groups are generally stable to radical attack, this decrease could be due to demethoxylation under the acidic conditions used during crosslinking.

According to the proposed mechanism, the activated OHphen is likely attack more reactive functional groups in lignin—those less stable than OCH_3_—leading to the formation of new ether bonds. This may explain the increase in H content in ether-type environments (50.3 vs. 12.7 wt.%, 3.00–3.46 ppm). However, it is important to note that this region also overlaps with the residual water signal, so the actual value may be slightly overestimated.

Moreover, a marked decrease in H associated with alkene groups (C=C) was observed (0.8 vs. 16.3 wt.%, 4.00–5.80 ppm). This could be explained by the activation of vinyl groups in the presence of the radical initiator, leading to radical coupling and chain propagation as illustrated in the following mechanism:

PPU–CH_2_–CH=CH_2_ → PPU–CH*–CH=CH_2_ (activation with CaCl_2_/H_2_O_2_)

PPU–CH*–CH=CH_2_ + PPU–CH_2_–CH=CH_2_ → PPU–CH_2_–CH=CH–CH_2_–CH*–CH_2_–PPU

This reaction would explain both the decrease in alkene signals and the increase in CH_2_-associated H (12.8 vs. 6.3 wt.%, 1.10–1.40 ppm). Other detected protons were attributed to CH_3_ groups (4.6 wt.%, 0.8–1.0 ppm) and miscellaneous environments (5.3 wt.%).

The ^31^P NMR results (Supplementary Material, Figure S20) indicated that all the OHphen and OHaliph functional groups reacted during crosslinking, as their presence in PolyActDLB was below the detection limit. Only a small amount of OHcarb (0.17 mmol/g) was detected, and this group is likely responsible for the low-intensity OH and C=O bands in the ATR-FTIR spectrum.

These results confirm the formation of a structurally distinct, thermally stable, and crosslinked aromatic material. The reactivity of depolymerized LB not only enhances functional group availability but also enables the design of lignin-based thermoset polymers with tailored properties.

## 3. Materials and Methods

### 3.1. Raw Material

LB and black liquor, both from Kraft pulping of *Eucalyptus globulus* wood, were provided by RAIZ—Forest and Paper Research Institute (Aveiro, Portugal). Both materials were stored in a cool, dry, and dark place.

KL from black liquor was recovered applying precipitation and washing. For this, black liquor was maintained at 45 °C for 1 h. Then, the pH was decreased down to 9.5 using a 6 M H_2_SO_4_ solution and was stirred for 1 h so that lignin precipitation occurred. The precipitate was vacuum-filtered and washed with acidic water at pH 3.0. The solid was dried under a vacuum.

Non-stabilized hydrogen peroxide aqueous solution (H_2_O_2_, 30% *w*/*v*, PanReac AppliChem, Darmstadt, Germany) was used for the depolymerization experiments. Sodium hydroxide pellets (NaOH, Merck, Rahway, NJ, USA) and sulfuric acid 96% (H_2_SO_4_, Merck, Rahway, NJ, USA) were used to adjust pH. Pyridine (Merck, Rahway, NJ, USA) and acetic anhydride (analytical reagent grade, Fluka, Buchs, Switzerland) were used for lignin acetylation. Hydrogen chloride (HCl, 1 M, Sigma-Aldrich, St. Louis, MO, USA) was used for the precipitation of acetylated lignin and lignin solubilized at alkaline pH. Iron (II) sulfate heptahydrate (FeSO_4_·7H_2_O, PanReac AppliChem, Darmstadt, Germany) was used as a catalyst during radical crosslinking. Calcium chloride dihydrate (CaCl_2_·2H_2_O, Sigma-Aldrich, St. Louis, MO, USA) and non-stabilized hydrogen peroxide aqueous solution (H_2_O_2_, 30% *w*/*v*, PanReac AppliChem, Darmstadt, Germany) were used for depolymerized lignin activation during crosslinking.

Tetrahydrofuran (THF, HPLC grade, Fisher chemical, Pittsburgh, PA, USA), dimethyl sulfoxide-d6 (DMSO-d_6_, for NMR, Thermo Scientific, Waltham, MA, USA), chloroform-d (for NMR, Thermo Scientific, Waltham, MA, USA), cholesterol (Sigma-Aldrich, St. Louis, MO, USA), chromium(III) 2,4-pentanedionate (Cr(acac)3, Sigma-Aldrich, St. Louis, MO, USA), pyridine anhydrous (Sigma-Aldrich, St. Louis, MO, USA), 2-chloro-4,4,5,5-tetramethyl-1,3,2-dioxaphospholane (TMDP, Sigma-Aldrich, St. Louis, MO, USA), and N-Methyl-N-(trimethylsilyl)trifluoroacetamide (MSTFA, 97%, Thermo Scientific, Waltham, MA, USA) were used as received for the characterization of lignin samples, depolymerized lignin, and the lignin-derived crosslinked matrix.

### 3.2. Oxidative Depolymerization of Lignin

Depolymerization experiments were conducted to study the effect of (1) temperature, (2) stirring, (3) pH, and (4) type of lignin, followed by the scale-up of the best conditions. The experiments were carried out in a 250 mL round-bottom glass reactor (Labbox LBG 3.3, Labbox Labware, Barcelona, Spain) equipped with magnetic stirring, cooling condenser, and reagents inlet. The temperature was maintained via an oil bath controlled by a heating stirring plate with a Pt1000 sensor (H20SQC, LBX instruments, Lexington, KY, USA).

In the study of temperature and stirring, LB was suspended at a high concentration (300 mg/mL) in a 30% (*w*/*v*) H_2_O_2_ solution in water, therefore using a 1:1 (*w*/*w*) H_2_O_2_:lignin ratio. The reagents were mixed to a homogeneous slurry resulting in a pH close to 1 due to the acidic character of H_2_O_2_ and the protonated character of LB. The reactions were carried out at 50, 60, and 70 °C, collecting periodic samples (Table 4). These samples, with a high concentration of solids, were dried at room temperature in Petri dishes prior to characterization. In this way, all the depolymerization components, both water-soluble and non-water-soluble, were recovered. The operation at different temperatures and times is associated with a distinct severity factor, first mentioned by Overend and Chornet (1987) [30]. This term is commonly used to describe the combined effects of temperature and time on a given reaction, expressed by the following equation:

logR_0_ = log[t (h) × exp((T (°C) − T_R_)/ω)], (1)

where logR_0_ is the severity factor, t is the reaction time (in hours), T is the temperature (°C), T_R_ is the reference temperature, and ω is a constant related to the activation energy of the specific process. In this work, the values T_R_ = 40 °C and ω = 5 were selected to better reflect the mild operating conditions (50–70 °C) and the slower reaction kinetics of the oxidative depolymerization process. A reduced ω value is consistent with literature values reported for processes such as enzymatic hydrolysis, where similar constants have been adopted [31].

The influence of stirring was studied through its suppression in a depolymerization experiment at 60 °C (LB-pH1-0rpm-60 °C).

The effect of pH was studied by depolymerization under alkaline conditions because at neutral pH H_2_O_2_ loses its oxidizing character [11]. LB was first dissolved in NaOH solution with a NaOH:LB ratio 0.6:1 (*w*/*w*). After that, the corresponding volume of 30% *w*/*v* H_2_O_2_ was added dropwise to maintain a 1:1 (*w*/*w*) H_2_O_2_:LB ratio. The resulting pH was ~10. Periodic samples were taken throughout the reaction. In this case, LB and the depolymerization products were soluble in the alkaline reaction medium. For the recovery of the samples, precipitation was carried out by decreasing the pH to 2 with a 0.1 M HCl. After precipitation, the samples were centrifuged, washed with distilled water, and dried under a vacuum prior to characterization. The recovered solid was characterized, while the reaction products soluble in acidic water were mostly lost during centrifugation and washing, together with the salts formed as a result of the change in the pH for the precipitation process.

The effect of lignin type was studied by comparing the same acid depolymerization process applied to KL versus LB. For this purpose, KL was mixed with the corresponding volume of 30% (*w*/*v*) H_2_O_2_ so that the ratio H_2_O_2_:KL (*w*/*w*) was 1. The result of the mixture was a homogeneous slurry whose pH was close to 5, proof of the higher alkaline character of KL versus LB. As a reference, depolymerization was carried out at these pH conditions, operating at 60 °C and taking periodic samples (KL-pH5-60 °C). To optimize the process and obtain a better comparison with LB, the process was repeated by adjusting pH to ~1, the same as with LB, by adding 1M H_2_SO_4_ (KL-pH1-60 °C). The reaction was carried out at 60 °C and periodic samples were taken and, after drying at room temperature, were characterized.

Lastly, the acidic oxidative depolymerization process was scaled up by selecting 60 °C as the temperature. In the first test, the reaction volume was increased by a factor of 4, using a round bottom glass reactor of the same type as in the previous experiments but with a 4 times larger volume (LB-pH1-60 °C-×4). Magnetic stirring and an oil bath for heating were employed, as in the case of previous experiments. Periodic samples were taken and dried at room temperature in larger Petri dishes. Then, a scale-up was carried out by increasing the volume by a factor of 25 (LB-pH1-60 °C-×25). This required the use of a 1 L glass reactor, coupled to a condenser, and with more precise temperature control thanks to a water jacket supplied by a thermal bath (Haake D1 L, Haake, Vreden, Germany). Stirring efficiency was enhanced by coupling a motorized mechanical stirrer (DLH, VELP Scientifica, Usmate Velate, Italy). In this case, no periodical samples were taken, and all the final solid was collected and, as in the previous cases, dried on a glass plate at room temperature. The recovered solid, including water-soluble and non-water-soluble compounds, was characterized.

**Figure 17:**
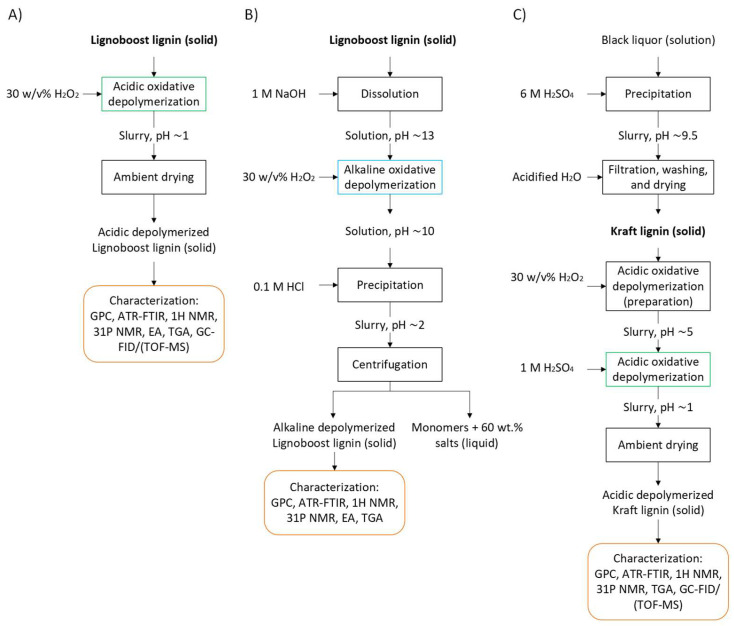
Block diagrams of the depolymerization processes: (**A**) acidic oxidative depolymerization of Lignoboost lignin, (**B**) alkaline oxidative depolymerization of Lignoboost lignin, and (**C**) acidic oxidative depolymerization of Kraft lignin.

Figure 17 presents a schematic overview of the three oxidative depolymerization workflows applied in this study: acidic oxidative depolymerization of LB, alkaline oxidative depolymerization of LB, and acidic oxidative depolymerization of KL.

### 3.3. Radical Crosslinking of the Depolymerized Lignin

The product derived from the acidic oxidative depolymerization scale-up (LB-pH1-60 °C-×25) was subjected to crosslinking using CaCl_2_/H_2_O_2_ redox system as a radical initiator activating the OH groups of the sample. The solid used for crosslinking consisted of both the water-soluble and water-insoluble fractions recovered after drying the entire depolymerization slurry at room temperature, i.e., the same solid that was subjected to characterization. The reaction was carried out in a 250 mL three-neck Schlenk flask reactor (Labbox LBG 3.3, Spain) equipped with magnetic stirring, cooling condenser, N_2_ inlet, and reagents inlet. The temperature was maintained with an oil bath controlled by a heating-stirring plate with a Pt1000 sensor (LBX H20SQC, LBX instruments). The reaction took place at 55 °C using water as the reaction medium. First, 1.362 g of CaCl_2_·2H_2_O (0.33:1 *w*/*w* CaCl_2_:depolymerized lignin) was dissolved in 14 mL of distilled water. Then, 3.0 g of depolymerized lignin (LB-pH1-60 °C-×25) was added. The mixture was kept under magnetic stirring for 5 min under N_2_ atmosphere. After that time, 0.566 mL of 30% (*w*/*v*) H_2_O_2_ (5.5:1 (*w*/*v*) depolymerized lignin:H_2_O_2_) was added. The mixture was kept under stirring and N_2_ atmosphere for 2 h. After that time, the pH was adjusted to 3.0 with a NaOH solution and FeSO_4_ (0.7 ppm of depolymerized lignin) previously dissolved in water was added. The reaction was maintained for 4 h under these conditions. The final mixture was vacuum-filtered and washed with distilled water. The washed solid was vacuum-dried overnight.

### 3.4. Characterization of Lignoboost Lignin (LB), Kraft Lignin (KL), Depolymerized Lignin Samples, and Lignin-Derived Crosslinked Matrix

The molecular weight distribution of LB, KL, and dried depolymerized lignin samples was determined by applying gel permeation chromatography (GPC) using a column (KF-803L, Shodex, Tokyo, Japan) protected by a pre-column (KF-G 4A, Shodex, Tokyo, Japan), maintained at 40 °C. THF (HPLC grade) at a flow rate of 1 mL/min was used as a mobile phase. The molecular weight distribution was determined using a UV detector (UV-4070, Jasco, Tokyo, Japan) at 280 nm and the system was calibrated using eight polystyrene standards (266–62,500 Da) from Agilent Technologies (Santa Clara, CA, USA) and one ethylbenzene standard (106 Da) from LGC Ehrenstorfer (Augsburg, Germany). LB and depolymerized lignin samples were soluble in THF. On the contrary, KL and the samples derived from experiment KL-pH5-60 °C required acetylation, performed according to the method described by Maitz et al. (2020) [32]. 0.5 g of each sample was dissolved in 3 mL of acetic anhydride and 3 mL of pyridine. The acetylation took place in an Anton Paar Monowave 50 (Anton Paar, Graz, Austria), at 150 °C for 1 min. The product of the reaction was precipitated using 1% (*w*/*v*) HCl, centrifuge, washed with distilled water to neutral pH, and dried under vacuum at 40 °C. The acetylated dried solid, as well as the other lignin samples, were dissolved in THF for GPC analysis at a concentration of 1 mg/mL and were filtered before analysis using 0.45 µm PTFE filters (Merck Millipore, Burlington, MA, USA).

The proportion of monomers in each sample was estimated following the method previously described by Ramos-Andrés et al. (2022) [33]. The detector intensity, assumed to be proportional to the analyte concentration, was used to integrate the chromatogram, and the area under the molecular weight distribution curve was divided into different molecular weight groups: monomers (<200 g/mol), dimers-trimers (201–873 g/mol), tetramers–decamers (874–2910 g/mol), and polymeric fraction (>2911 g/mol). The proportion of each group in the sample was calculated as the area in the corresponding region relative to the total area under the curve.

The lignin-derived crosslinked matrix was not soluble in THF. The GPC analysis was performed using two GPC columns in series (GRAM 1000 Å and GRAM 30 Å, PSS Polymer Standards Service, Mainz, Germany) protected by a pre-column (GRAM, 10 μm, PSS Polymer Standards Service, Mainz, Germany) and maintained at 70 °C. A mobile phase of DMF/0.05 M LiCl was employed at a flow rate of 0.6 mL/min. A UV detector was utilized to observe the molecular weight distribution, while a RI detector was used to determine the weight-average molecular weight (Mw) and the polydispersity index (Ð). The system was calibrated using poly (methyl methacrylate) (PMMA) standards (PSS Polymer Standards Service, Mainz, Germany). LB and the product of the experiment LB-pH1-60 °C-×25 were also analyzed by this method for comparison purposes. All the samples were dissolved in DMF/0.05 M LiCl at a concentration of 1 mg/mL and were filtered before analysis using 0.45 µm PTFE filters (Merck Millipore, Burlington, MA, USA).

Thermogravimetric analysis (TGA) and derivative thermogravimetry (DTG) of LB, KL, depolymerized lignin samples, and lignin-derived crosslinked matrix were carried out using a STA7200 TGA/SDTA analyzer (Hitachi High-Tech Science Corporation, Tokyo, Japan). Approximately 5 mg of each sample were heated under a N_2_ atmosphere (200 mL/min) at a rate of 10 °C/min from 35 to 600 °C.

The elemental analysis (EA) of LB and depolymerized samples from experiments LB-pH1-50, LB-pH1-60, LB-pH1-70, LB-pH1-0rpm-60, and LB-pH10-60 was performed with an EMA 502 elemental analyzer (Elemental Microanalysis Ltd., Okehampton, UK) to assess the carbon, hydrogen, nitrogen and sulfur content. The oxygen content was estimated by subtracting the other percentages from 100%. The empirical formula of the PPU was determined from the results of EA and the content of methoxy groups (OCH_3_) was determined using ^1^H NMR according to the method described by Sameni et al. (2016) [34].

The functional groups of LB, KL, dried depolymerized lignin samples, and lignin-derived crosslinked matrix were studied by conducting attenuated total reflectance (ATR)—Fourier transform infrared spectroscopy (FTIR) on a Spectrum Two FT-IR spectrometer (PerkinElmer, Waltham, MA, USA) equipped with a universal ATR (UATR) accessory. Approximately 5 mg of the solid sample was placed directly onto the diamond ATR crystal and pressed with a flat-tip anvil. Spectra were collected in the wavenumber range of 4000–400 cm^−1^, with a resolution of 4 cm^−1^ and 16 scans of data accumulation.

A Bruker Avance III 400 spectrometer (Bruker, Karlsruhe, Germany) was used to conduct ^1^H NMR and ^31^P NMR analysis. For ^1^H NMR, 50 mg of dry samples were dissolved in 1 mL of DMSO-d_6_ and sonicated for 60 min. ^1^H NMR was used to determine the content of aromatic hydrogen, methoxy groups (OCH_3_) and hydrogen in alkene groups (C=C) in LB, KL, depolymerized lignin samples, and lignin-derived crosslinked matrix. ^31^P NMR was performed following the methodology described by Meng et al. (2019) [35], and it was used to determine the content of phenolic, aliphatic and carboxylic hydroxyl groups (OH) in LB, KL, and depolymerized lignin samples.

Before GC-FID/(TOF-MS) analysis, the solid samples of depolymerized lignin from experiments LB-pH1-50, LB-pH1-60, LB-pH1-70, KL-pH1-60 °C were derivatized. To do this, 50 mg of the sample was dissolved in 1 mL of anhydrous pyridine under stirring, and then filtered using 0.45 µM PTFE filters (Merck Millipore, Burlington, MA, USA) before being introduced into a GC vial. Next, 0.5 mL of the derivatizing agent, MSTFA, was added to the GC vial. The derivatization reaction was carried out at 80 °C for 30 min. Afterward, 0.5 mL of anhydrous dichloromethane was added to the vial to dilute the sample. The monomers of the derivatized depolymerized lignin samples were identified using a two-column gas chromatography (GC) system. A volume of 1.0 µL of the sample was injected in split mode (1:10) into the GC × GC quadjet system (LECO Corporation, Saint Joseph, MI, USA), with the injector temperature set at 280 °C. The carrier gas flow rate was 1.8 mL/min. The chromatographic system consisted of a primary column (Rxi-5ms, 30 m length, 0.25 mm inner diameter, and 0.25 µm film thickness, Restek Corporation, Bellefonte, PA, USA), connected to a secondary column (Rxi-17 Sil MS, 1.6 m length, 0.25 mm inner diameter, and 0.25 µm film thickness, Restek Corporation, Bellefonte, PA, USA). The primary oven temperature was initially set at 40 °C for 3 min, followed by a heating ramp of 10 °C/min to 300 °C, where it remained for 11 min. The system was operated in a one-dimensional mode, with both Flame Ionization Detector (FID) and Time of Flight (TOF) Mass Spectrometry detectors used for identification. The FID temperature was set to 300 °C, and the TOF transfer line temperature was set to 280 °C, with an ionization energy of 70 eV.

## 4. Conclusions

Lignoboost lignin (LB) was successfully depolymerized and functionalized using oxidative depolymerization with H_2_O_2_ under inherently acidic conditions, without the use of organic solvents or catalysts. The effects of temperature, time, and stirring were studied. The LB was suspended at a high concentration (300 mg/mL) and its protonated character provided sufficient acidity for the depolymerization process. Conditions of 60 °C and 3 h were enough to achieve good results, with a major fraction of aromatic dimers-trimers (68.6 wt.%) highly functionalized (2.75 mmol/g OHphen, 3.58 mmol/g OHcarb, 19.5 wt.% of H in -CH=CH-), as well as aliphatic dicarboxylic acids (53.4 wt.% of the monomers). The effect of pH was studied by comparing alkaline conditions (where LB is dissolved in the medium) to acidic conditions. Alkaline conditions partially oxidized LB but did not allow for adequate depolymerization or functionalization. The versatility of the process was demonstrated by applying the optimal acidic conditions to a less pure lignin, Kraft lignin (KL), which required the addition of external acid but still yielded satisfactory results. The process was scaled up (×25) for LB, incorporating improvements in temperature control and mass transfer, resulting in a significant reduction in Mw and Đ from 979 g/mol and 1.5 to 464 g/mol and 1.3, respectively. The scaled-up solid product was subjected to radical crosslinking, leveraging the reactivity of OHphen and alkene groups, which can be activated to form a polyether-type crosslinked structure with high aromaticity and superior thermal stability compared to LB (54.2 wt.% versus 36.1 wt.% residual mass at 600 °C). Overall, this work demonstrates that selective depolymerization towards functionalized low-molecular-weight lignin oligomers and aliphatic dicarboxylic acids is a viable and valuable strategy, as these compounds present significant opportunities for applications in bio-based polymers, adhesives, coatings, and advanced materials. Future work will focus on tailoring the structural features of the oligomers and exploring their incorporation into functional materials through copolymerization and advanced crosslinking strategies.

## Figures and Tables

**Table 1:** Elemental analysis and empirical formula in Lignoboost lignin after acidic oxidative depolymerization under different severity factors.

Sample	logR_0_	C	H	O	S	N	PPU Empirical Formula	Mw_PPU_ (g/mol)
LB	-	57.90 ± 0.03	5.55 ± 0.02	32.86 ± 0.06	3.10 ± 0.24	0.52 ± 0.33	C_9_H_5.944_O_2.461_S_0.228_N_0.087_(OCH_3_)_2.372_	235.69
50 °C, 7 h	1.71	50.73 ± 0.19	4.71 ± 0.02	39.41 ± 0.34	4.66 ± 0.28	0.51 ± 0.17	C_9_H_6.083_O_4.373_S_0.382_N_0.095_(OCH_3_)_2.085_	262.24
60 °C, 3 h	2.21	48.56 ± 0.16	4.36 ± 0.02	41.90 ± 0.26	4.57 ± 0.21	0.62 ± 0.11	C_9_H_7.102_O_5.350_S_0.365_N_0.113_(OCH_3_)_1.343_	255.60
70 °C, 2 h	2.91	46.48 ± 0.09	4.71 ± 0.20	43.46 ± 0.12	4.84 ± 0.25	0.52 ± 0.12	C_9_H_8.254_O_5.860_S_0.410_N_0.101_(OCH_3_)_1.508_	271.28

LB: Lignoboost lignin; logR_0_: severity factor; PPU: phenylpropane unit; Mw_PPU_: molecular weight of the phenylpropane unit.

**Table 2:** GC-FID/(TOF-MS) identification of monomers derived from acidic oxidative depolymerization in Lignoboost lignin and Kraft lignin.

	Formula	Mw (g/mol)	LB 50 °C, 7 h logR_0_ 1.71	LB 60 °C, 3 h logR_0_ 2.21	LB 70 °C, 2 h logR_0_ 2.91	KL 60 °C, 3 h logR_0_ 2.21
**Alcohols**	-	-	2.01%	2.04%	1.44%	1.33%
Ethylene glycol	C_2_H_6_O_2_	62.08	2.01%	2.04%	1.44%	1.33%
**Hydroxycarboxylic acids**	-	-	14.73%	14.73%	16.93%	19.21%
Lactic acid	C_3_H_6_O_3_	90.09	0.72%	0.74%	0.53%	4.24%
Glycolic acid	C_2_H_4_O_3_	76.06	9.15%	9.27%	11.40%	8.94%
2-Hydroxybutyric acid	C_4_H_8_O_3_	104.12	0.43%	0.44%	0.22%	3.10%
Glyceric acid	C_3_H_6_O_4_	106.09	3.03%	3.07%	3.15%	1.95%
Malic acid	C_4_H_6_O_5_	134.10	1.40%	1.42%	1.63%	0.97%
**Dicarboxylic acids**	-	-	50.28%	53.40%	56.21%	29.36%
Oxalic acid	C_2_H_2_O_4_	90.04	27.14%	27.52%	15.72%	8.88%
Propanedioic acid	C_3_H_4_O_4_	104.07	19.96%	22.65%	36.26%	18.23%
Succinic acid	C_4_H_6_O_4_	118.10	1.76%	1.79%	2.19%	1.26%
Ethylmalonic acid	C_5_H_8_O_4_	132.13	1.42%	1.44%	2.03%	0.99%
**Esters**	-	-	1.65%	1.68%	11.28%	6.43%
Methyl 2-hydroxypropanoate	C_4_H_8_O_3_	104.12	0.18%	0.18%	1.88%	0.99%
Monomethyl succinate	C_5_H_8_O_4_	132.13	n.d.	n.d.	3.35%	1.28%
Methyl 2-hydroxyethyl malonate	C_6_H_10_O_5_	162.16	n.d.	n.d.	4.88%	2.71%
Butyl 6-methylheptanoate	C_12_H_2__4_O_2_	200.36	1.47%	1.49%	1.17%	1.45%
**Lactones**	-	-	0.54%	0.55%	2.39%	2.69%
3-Hydroxy-3-hydroxymethyl-dihydro-2(3H)-furanone	C_5_H_8_O_4_	132.13	0.39%	0.40%	2.08%	0.55%
2,3,4,5-Tetrahydroxypentanoic acid-1,4-lactone	C_5_H_8_O_6_	164.11	n.d.	n.d.	n.d.	0.42%
Erythrono-1,4-lactone	C_4_H_6_O_4_	118.10	0.15%	0.15%	0.31%	1.32%
D-Erythro-Pentonic Acid, γ-Lactone	C_5_H_8_O_5_	148.11	n.d.	n.d.	n.d.	0.41%
**Monosaccharides**	-	-	12.16%	10.87%	2.10%	29.91%
D-Arabinopyranose	C_5_H_10_O_5_	150.15	0.78%	0.79%	0.00%	1.40%
β-Arabinopyranose	C_5_H_10_O_5_	150.15	0.00%	0.00%	0.72%	1.58%
D-ribose	C_5_H_10_O_5_	150.15	1.44%	0.00%	0.55%	0.99%
D-xylose	C_5_H_10_O_5_	150.15	4.76%	4.83%	0.42%	11.95%
β-D(-)-Lyxopyranose	C_5_H_10_O_5_	150.15	5.18%	5.25%	0.41%	11.56%
Methyl xylopyranoside	C_6_H_12_O_5_	164.18	n.d.	n.d.	n.d.	2.43%
**Aromatics**	-	-	12.89%	12.57%	2.73%	2.48%
2,6-Dimethoxyhydroquinone	C_6_H_4_(OH)(OCH_3_)_2_	170.18	0.50%	n.d.	0.19%	0.34%
Vanillic acid	C_6_H_4_(OH)(COOH)(OCH_3_)	168.16	1.91%	1.93%	0.74%	0.89%
Benzoic acid, 4-hydroxy-3,5-dimethoxy-	C_6_H_4_(OH)(COOH)(OCH_3_)_2_	198.19	0.32%	0.32%	n.d.	n.d.
Protocatechuic acid	C_6_H_4_(OH)(COOH)(OCH_3_)	154.13	0.70%	0.71%	0.39%	n.d.
Syringic acid	C_6_H_4_(OH)(COOH)(OCH_3_)_2_	198.19	3.38%	3.43%	0.30%	n.d.
Acetyl syringic acid	C_9_H_10_O_5_	240.23	1.16%	1.17%	0.40%	1.26%
Phthalic acid, di(2,3-dimethylphenyl) ester	C_6_H_4_(CO_2_R)_2_(OCH_3_)_2_	350.44	0.40%	0.40%	0.25%	n.d.
4,4′-Methylenedi-2,6-xylenol	C_14_H_14_O_2_	214.28	3.70%	3.75%	0.46%	n.d.
3-Hydroxy-7,8,2′,3′-tetramethoxyflavone	C_19_H_18_O_7_	358.37	0.83%	0.84%	n.d.	n.d.
**Other monomers**	-	-	5.74%	3.97%	6.93%	8.58%

Mw: molecular weight of the monomer; LB: Lignoboost lignin; logR_0_: severity factor; KL: Kraft lignin.

**Table 3:** Elemental analysis and empirical formula of PPU in Lignoboost lignin after acidic oxidative depolymerization: effect of stirring and pH.

Sample	C	H	O	S	N	PPU Empirical Formula	Mw_PPU_ (g/mol)
LB	57.9 ± 0.03	5.55 ± 0.02	32.86 ± 0.06	3.10 ± 0.24	0.52 ± 0.33	C_9_H_5.944_O_2.461_S_0.228_N_0.087_(OCH_3_)_2.372_	235.69
60 °C, 3 h, pH 1	48.56 ± 0.16	4.36 ± 0.02	41.90 ± 0.26	4.57 ± 0.21	0.62 ± 0.11	C_9_H_7.102_O_5.350_S_0.365_N_0.113_(OCH_3_)_1.343_	255.60
60 °C, 3 h, pH 1, no stirring	49.74 ± 0.08	4.58 ± 0.01	40.97 ± 0.14	4.23 ± 0.12	0.50 ± 0.06	C_9_H_6.956_O_4.959_S_0.337_N_0.091_(OCH_3_)_1.571_	255.06
60 °C, 3 h, pH 10	56.71 ± 0.22	5.17 ± 0.08	34.89 ± 0.18	2.62 ± 0.15	0.61 ± 0.26	C_9_H_4.837_O_2.732_S_0.202_N_0.107_(OCH_3_)_2.637_	246.26

LB: Lignoboost lignin; PPU: phenylpropane unit; Mw_PPU_: molecular weight of the phenylpropane unit.

**Table 4:** Oxidative depolymerization operating conditions.

Experiment	Type of Lignin	Lignin (g)	30% (*w*/*v*) H_2_O_2_ (mL)	Stirring	pH	T (°C)	Time (h)
LB-pH1-50 °C	LB	3	10	Magnetic	1	50	3, 5, 7
LB-pH1-60 °C					1	60	1, 2, 3
LB-pH1-70 °C					1	70	1, 2, 3
LB-pH10-60 °C					10	60	1, 2, 3
LB-pH1-0rpm-60 °C				No	1	60	1, 2, 3
KL-pH5-60 °C	KL	3	10	Magnetic	5	60	1, 2, 3
KL-pH1-60 °C					1		
LB-pH1-60 °C-×4	LB	12	40	Magnetic	1	60	1, 2, 3
LB-pH1-60 °C-×25		75	250	Mechanical			3

LB: Lignoboost lignin; KL: Kraft lignin.

## Data Availability

Data will be made available on request.

## Supplementary Materials

The following supporting information can be downloaded at https://www.mdpi.com/article/10.3390/ijms26104872/s1.

## Abbreviations

The following abbreviations are used in this manuscript:

LB	Lignoboost lignin
KL	Kraft lignin
DLB	Depolymerized Lignoboost lignin
DP	Degree of polymerization
PolyActDLB	Crosslinked polymer derived from DLB
PPU	Phenylpropane unit
Mw	Weight-average molecular weight
Đ	Dispersity index (Mw/Mn)
logR_0_	Logarithmic severity factor
OHphen	Phenolic hydroxyl group
OHaliph	Aliphatic hydroxyl group
OHcarb	Carboxylic hydroxyl group
OCH_3_	Methoxy group
C=C/-CH=CH-	Aliphatic carbon-carbon double bond (alkene)
C=O	Carbonyl group (in ketones, acids, esters, etc.)
C-O-C	Ether linkage
THF	Tetrahydrofuran
DMSO-d_6_	Deuterated dimethyl sulfoxide
MSTFA	N-Methyl-N-(trimethylsilyl)trifluoroacetamide (derivatizing agent)
TMDP	2-Chloro-4,4,5,5-tetramethyl-1,3,2-dioxaphospholane (for ^31^P NMR)
^1^H NMR	Proton nuclear magnetic resonance spectroscopy
^31^P NMR	Phosphorus-31 nuclear magnetic resonance spectroscopy
ATR-FTIR	Attenuated total reflectance Fourier-transform infrared spectroscopy
GPC	Gel permeation chromatography
RI	Refractive index
UV	Ultraviolet
EA	Elemental Analysis
TGA/DTG	Thermogravimetric analysis/Derivative thermogravimetry
DSC	Differential scanning calorimetry
GC-FID/(TOF-MS)	Gas chromatography with flame ionization detector and time-of-flight mass spectrometry
