# Supplementary material for: Acidic Oxidative Depolymerization Towards Functionalized Low-Molecular-Weight Lignin and High-Value-Added Aliphatic Monomers: Operating Conditions, Scale-Up, and Crosslinking
Source: Int J Mol Sci. 2025 May 19;26(10):4872. doi: 10.3390/ijms26104872 (PMC12112072; doi:10.3390/ijms26104872)
Supplement: Supplementary file 1 [file ijms-26-04872-s001.zip › ijms-3587641-supplementary.pdf]

Supplementary information

Acidic oxidative depolymerization towards functionalized low molecular weight lignin and high value-added aliphatic monomers: operating conditions, scale-up, and cross-linking

Marta C. Lourenço, Talita Nascimento, Pedro José Sanches Filho, Ana C. Marques and Marta Ramos-Andrés

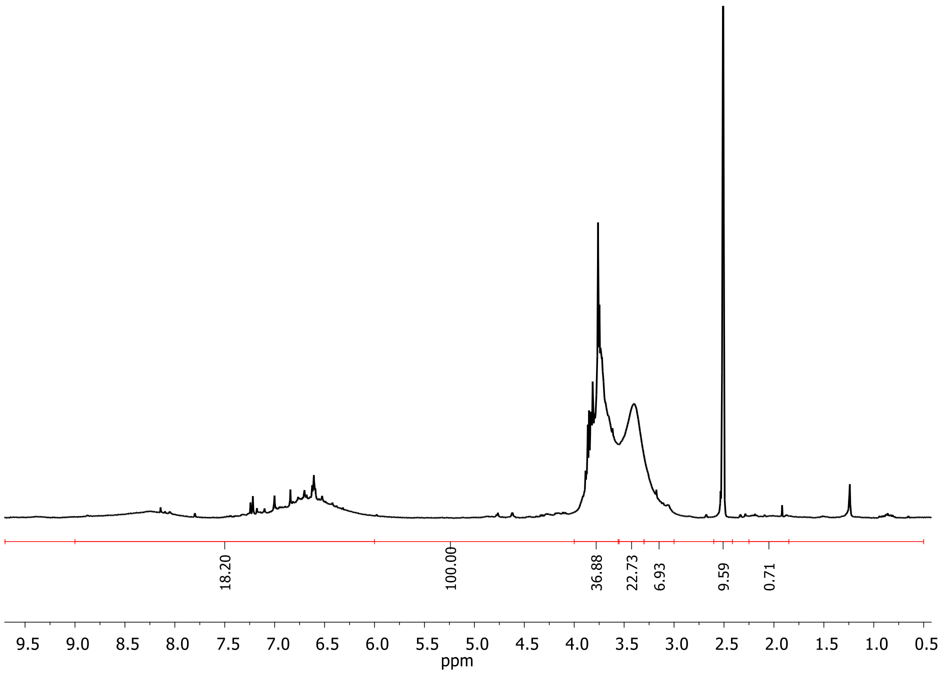

Figure S1. ^{1}H NMR spectrum of LB.

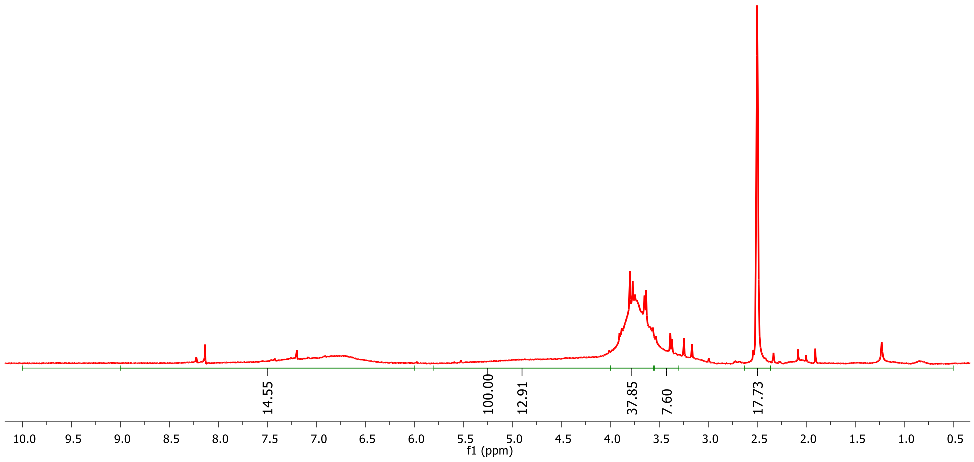

Figure S2. ^{1}H NMR spectrum of LB-pH1-50°C (7 h).

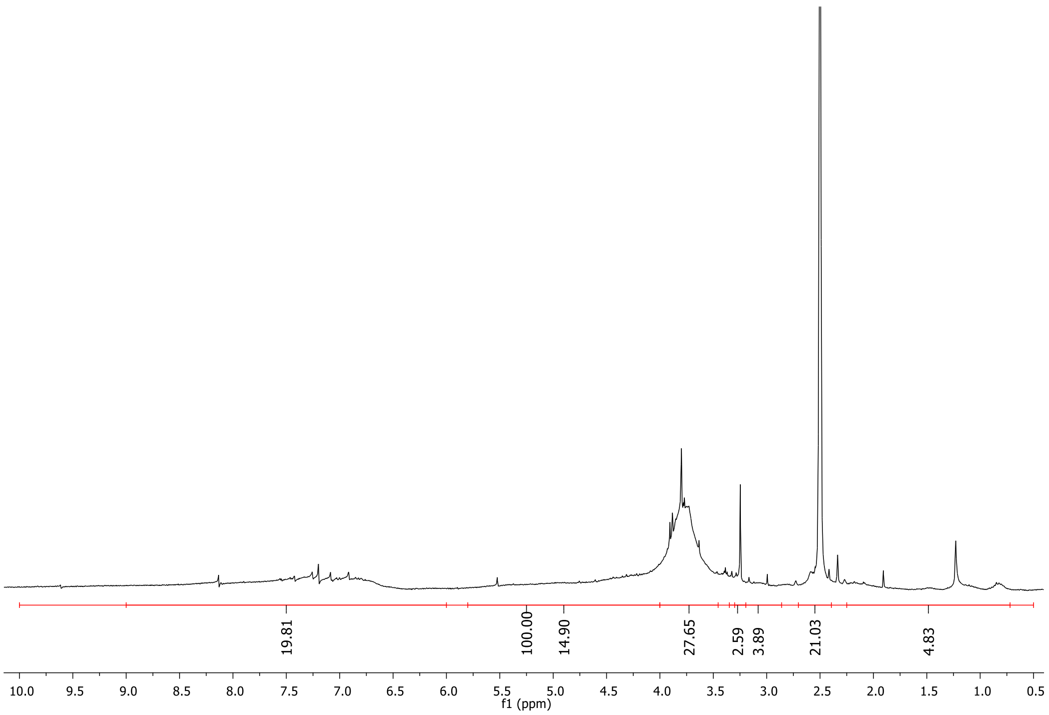

Figure S3. ^{1}H NMR spectrum of LB-pH1-60°C (3 h).

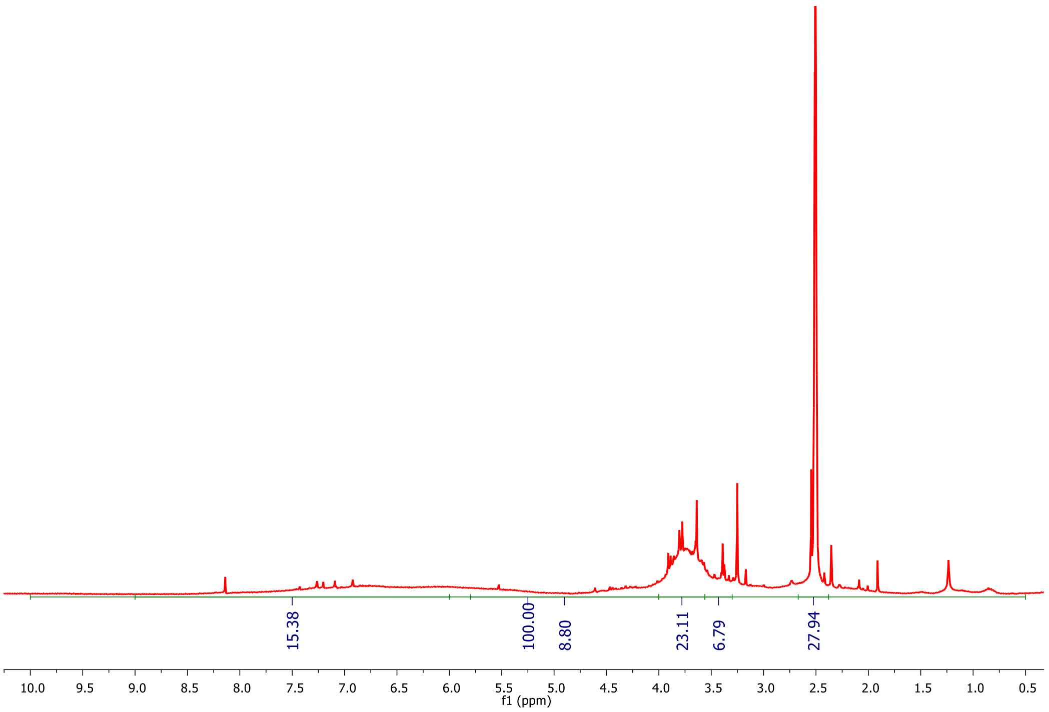

Figure S4. ^{1}H NMR spectrum of LB-pH1-70°C (2 h).

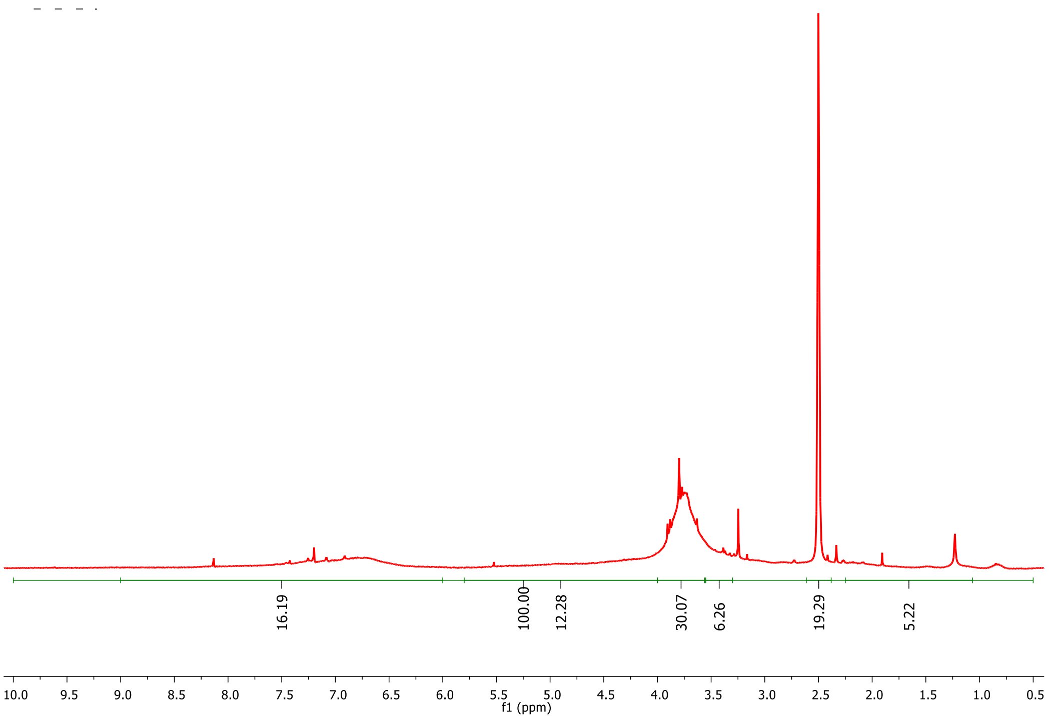

Figure S5. ^{1}H NMR spectrum of LB-pH1-0rpm-60°C (3 h).

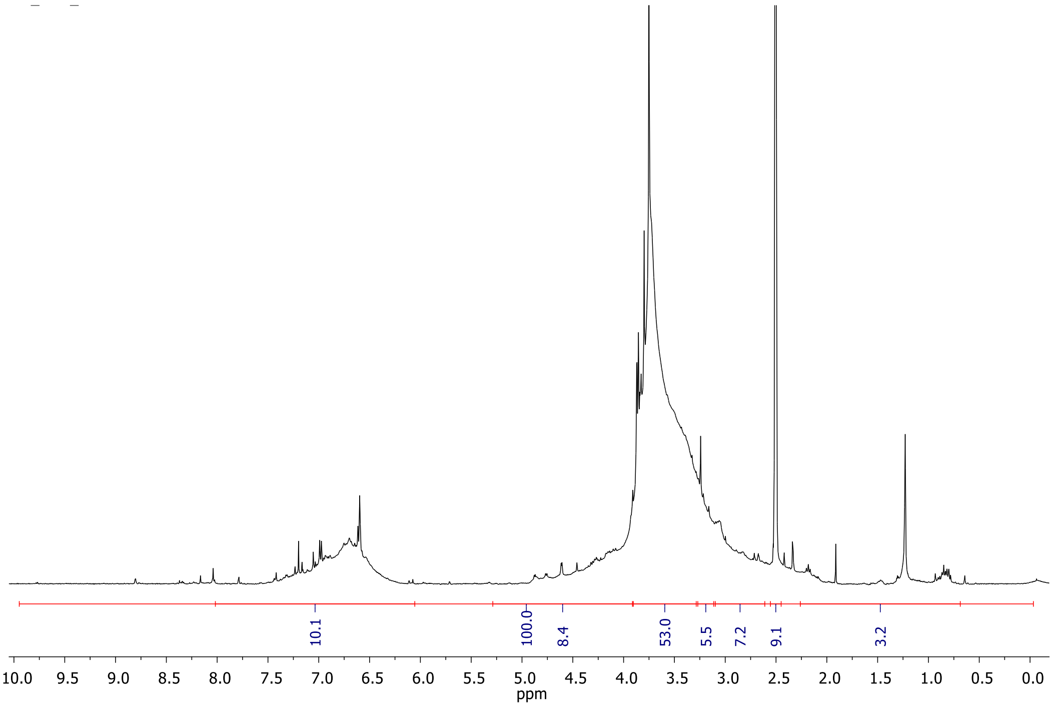

Figure S6. ^{1}H NMR spectrum of LB-pH10-60°C (3 h).

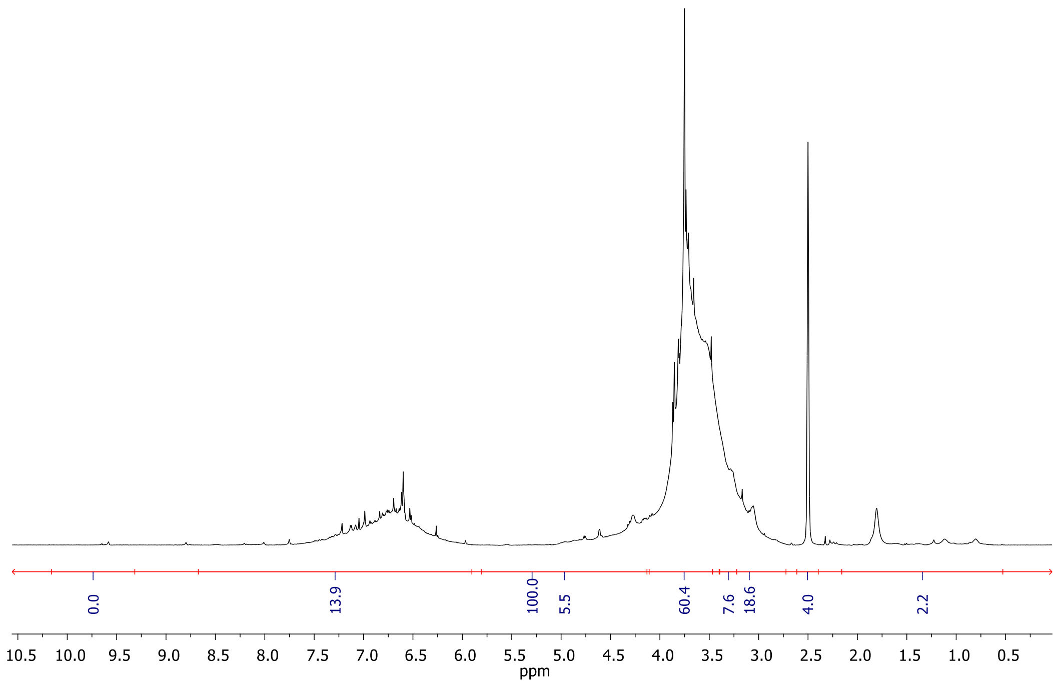

Figure S7. ^{1}H NMR spectrum of KL.

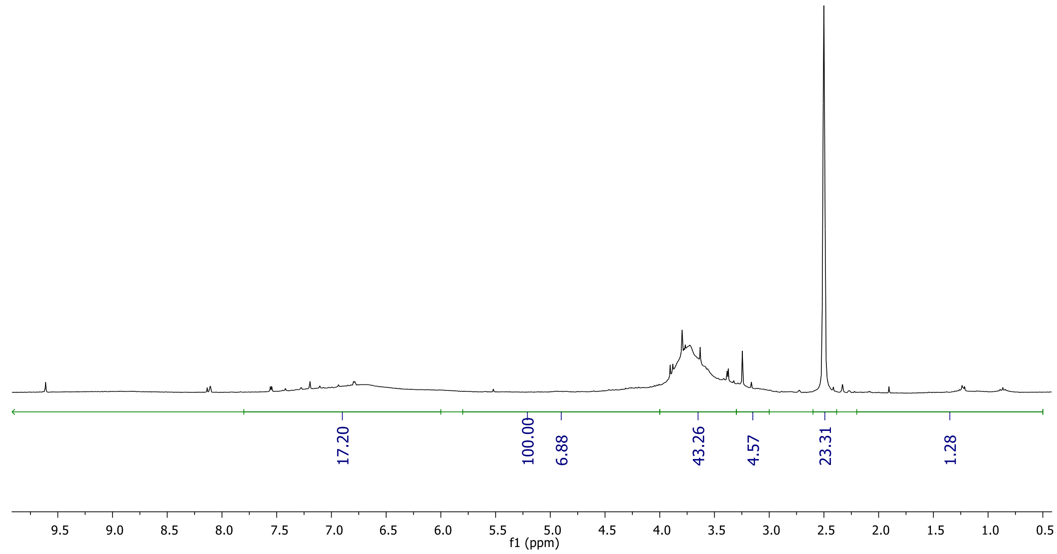

Figure S8. ^{1}H NMR spectrum of KL-pH1-60°C (3 h).

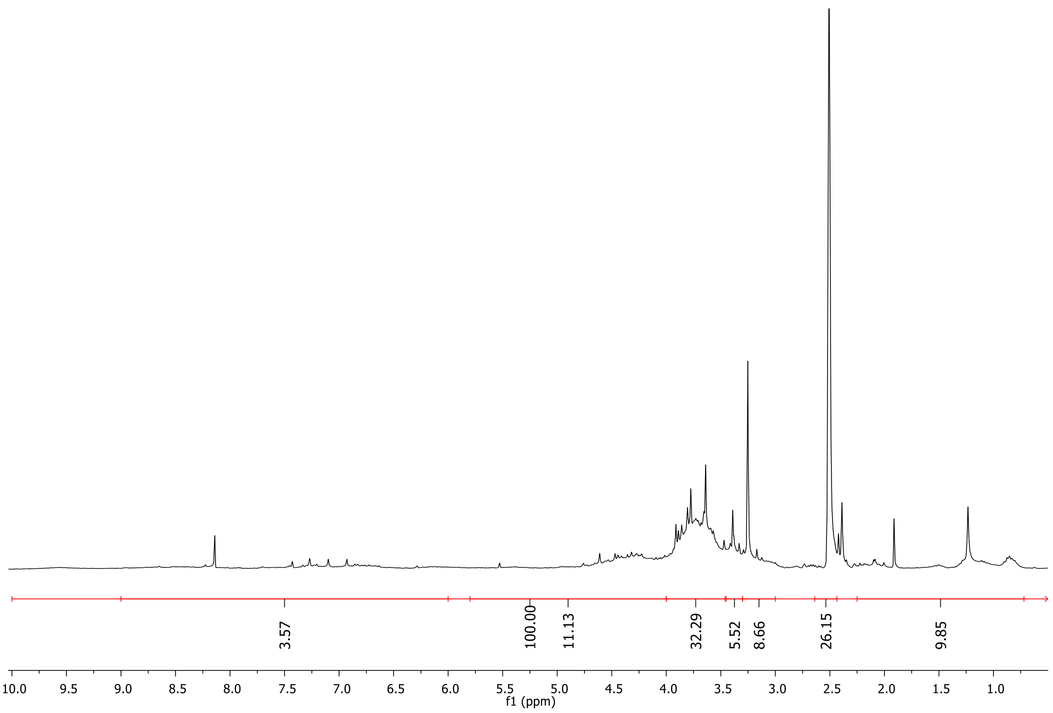

Figure S9. ^{1}H NMR spectrum of LB-pH1-60°C-×25.

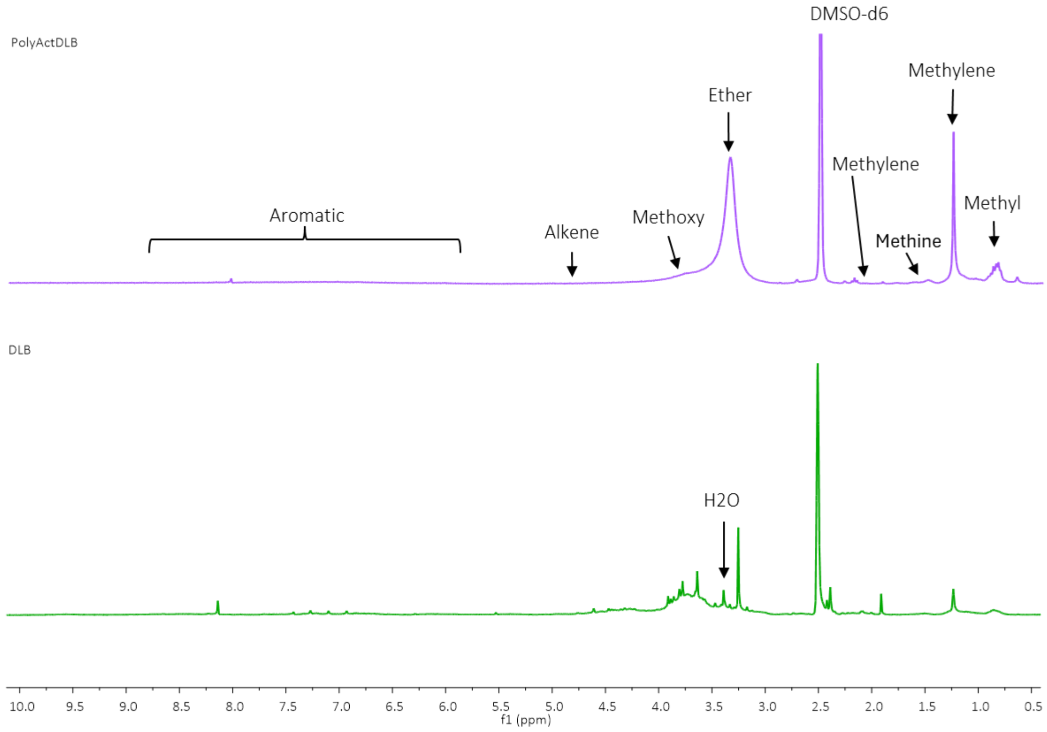

Figure S10. ^{1}H NMR spectrum of PolyActDLB and DLB.

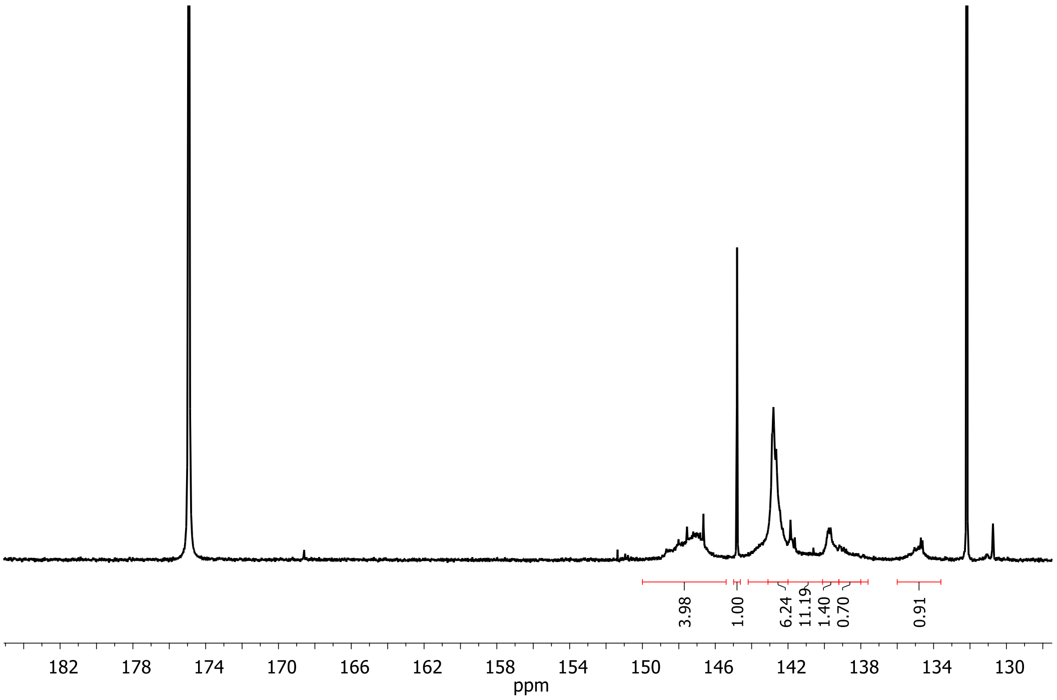

Figure S11. ^{31}P NMR spectrum of LB.

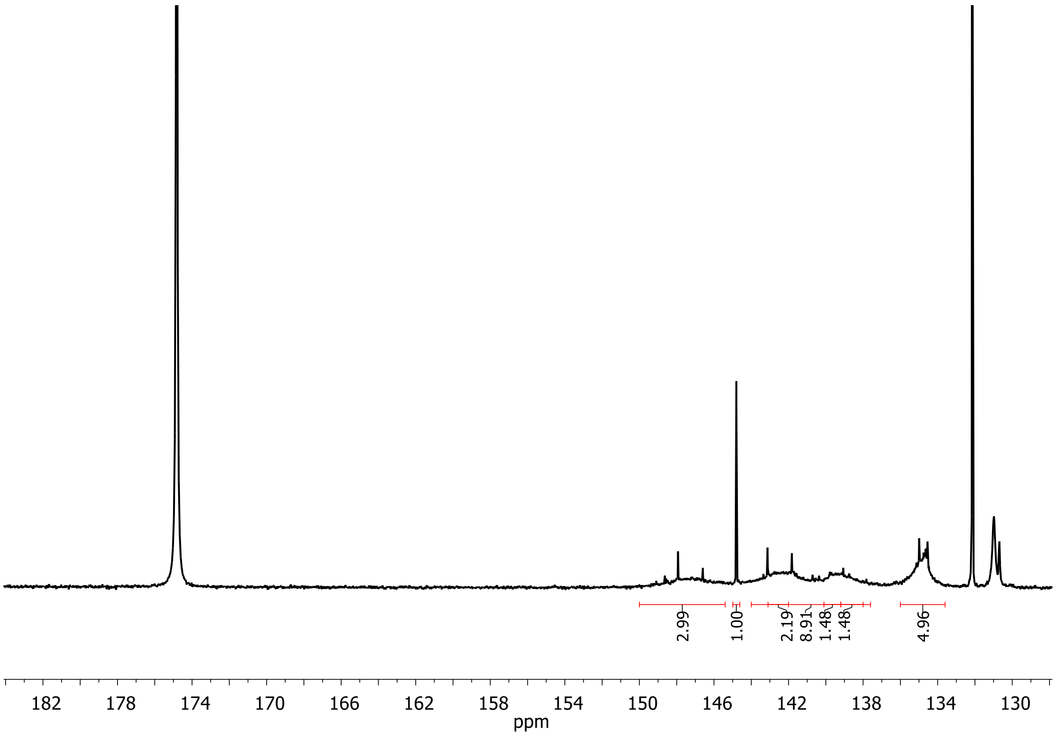

Figure S12. ^{31}P NMR spectrum of LB-pH1-50°C (7 h).

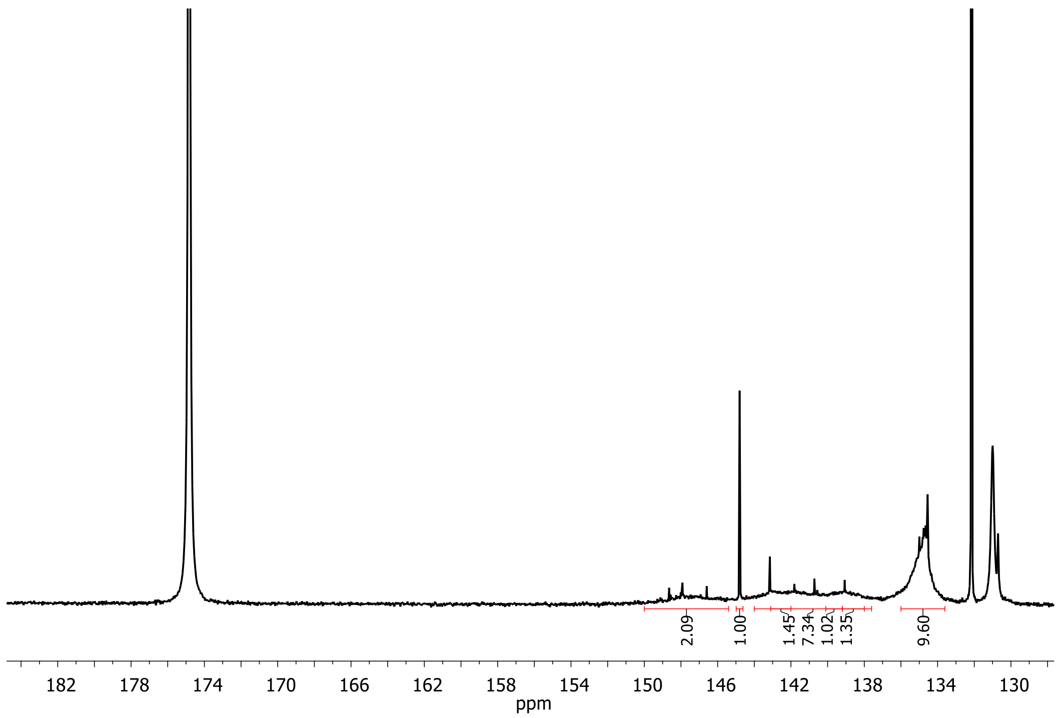

Figure S13. ^{31}P NMR spectrum of LB-pH1-60°C (3 h).

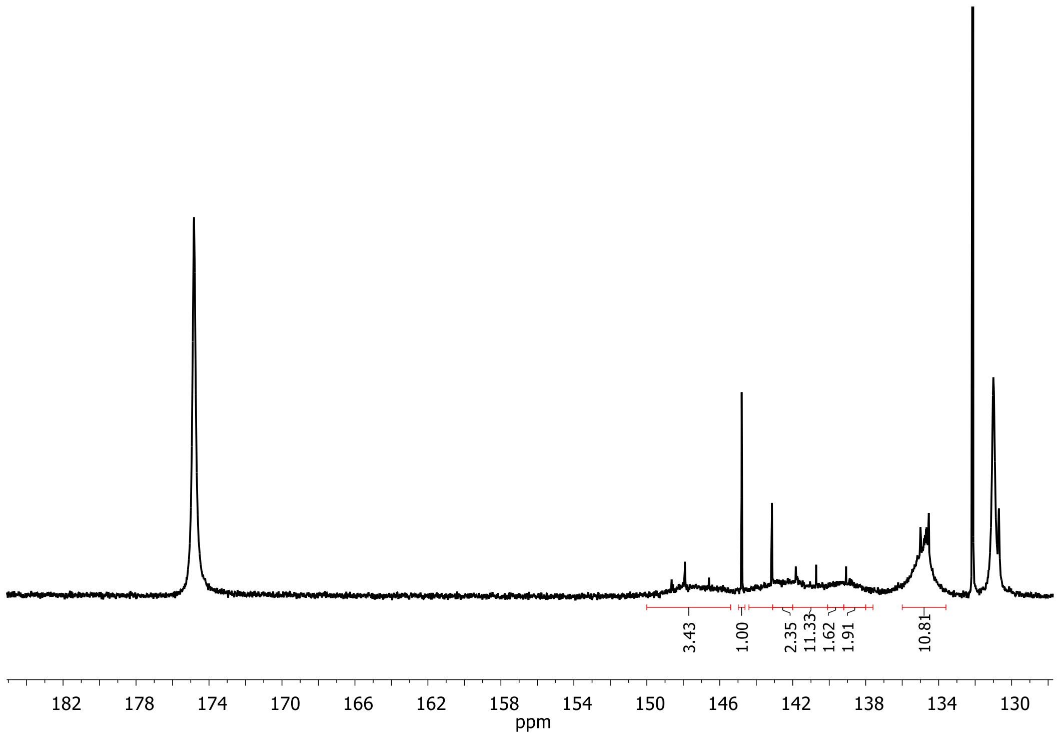

Figure S14. ^{31}P NMR spectrum of LB-pH1-70°C (2 h).

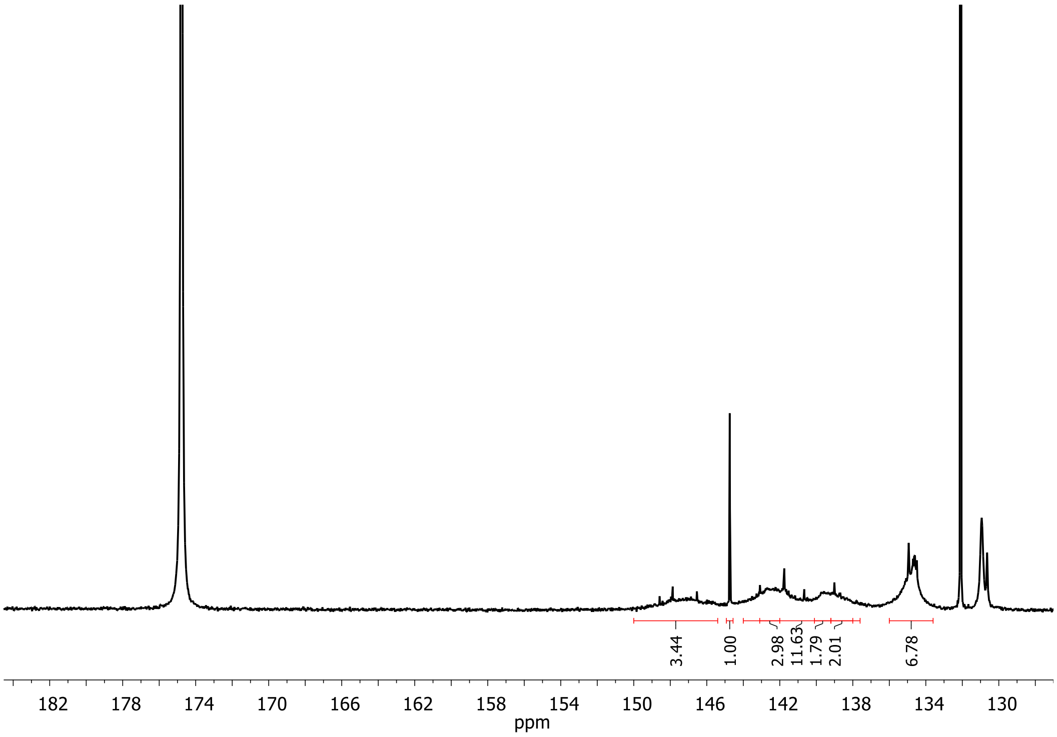

Figure S15. ^{31}P NMR spectrum of LB-pH1-0rpm-60°C (3 h).

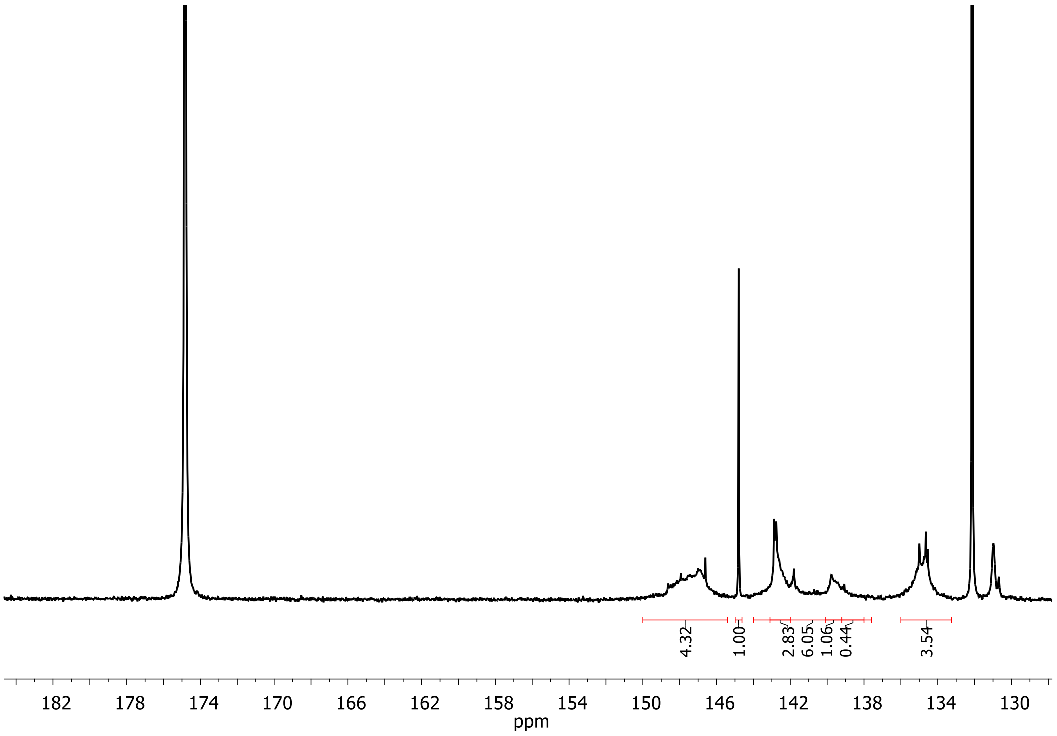

Figure S16. ^{31}P NMR spectrum of LB-pH10-60°C (3 h).

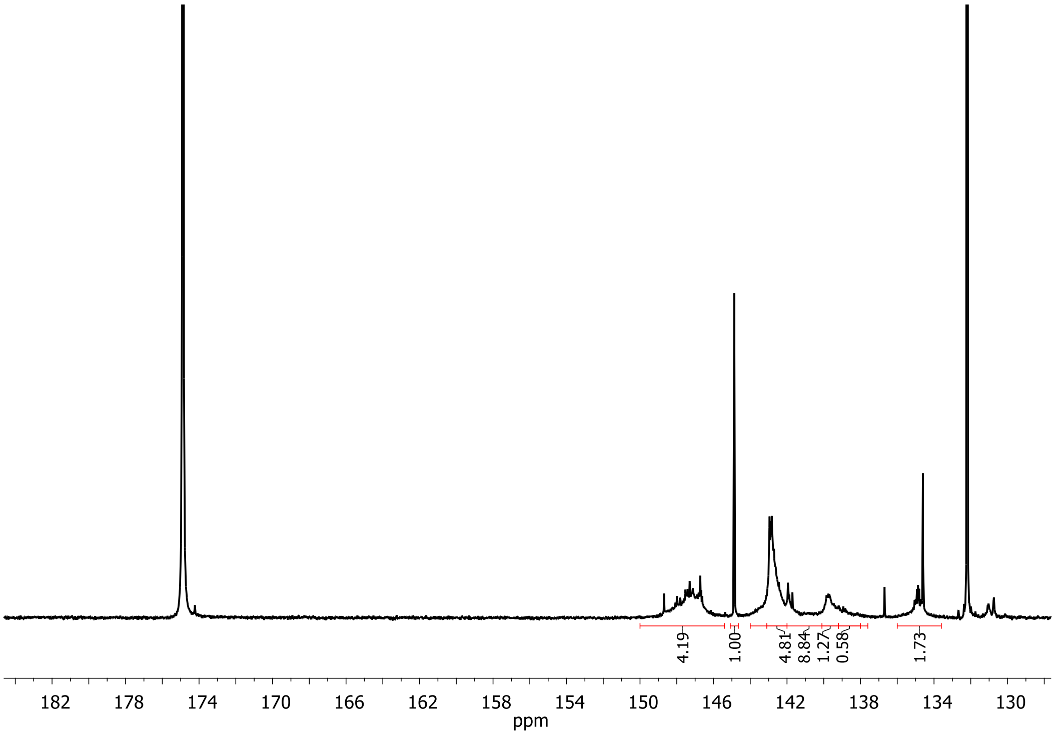

Figure S17. ^{31}P NMR spectrum of KL.

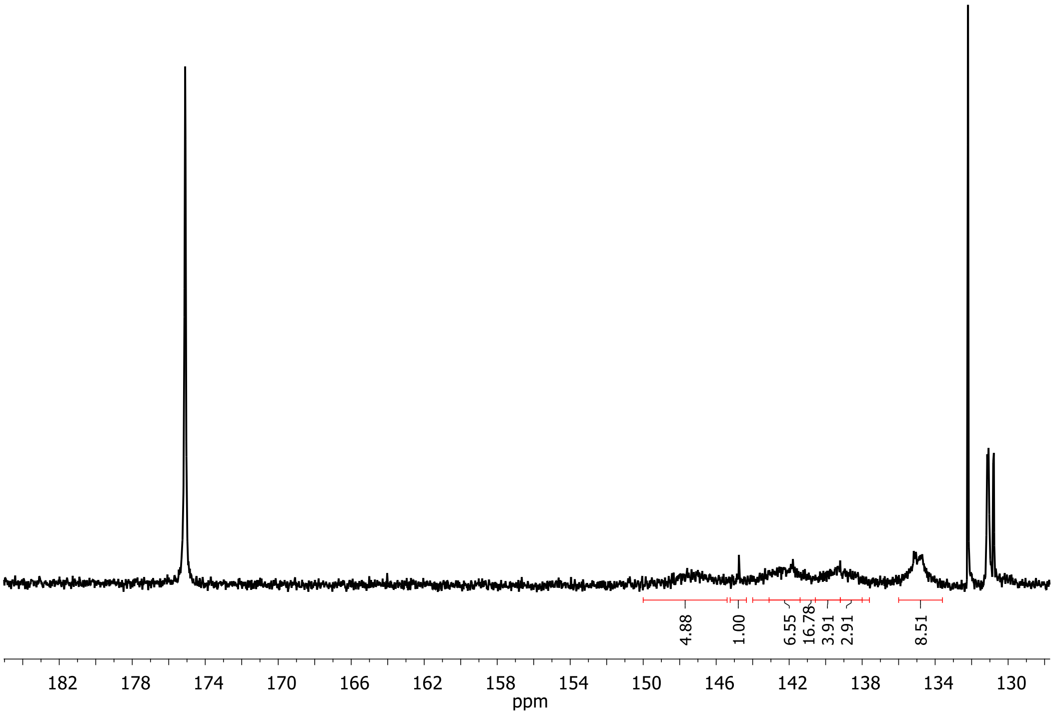

Figure S18. ^{31}P NMR spectrum of KL-pH1-60°C (3 h).

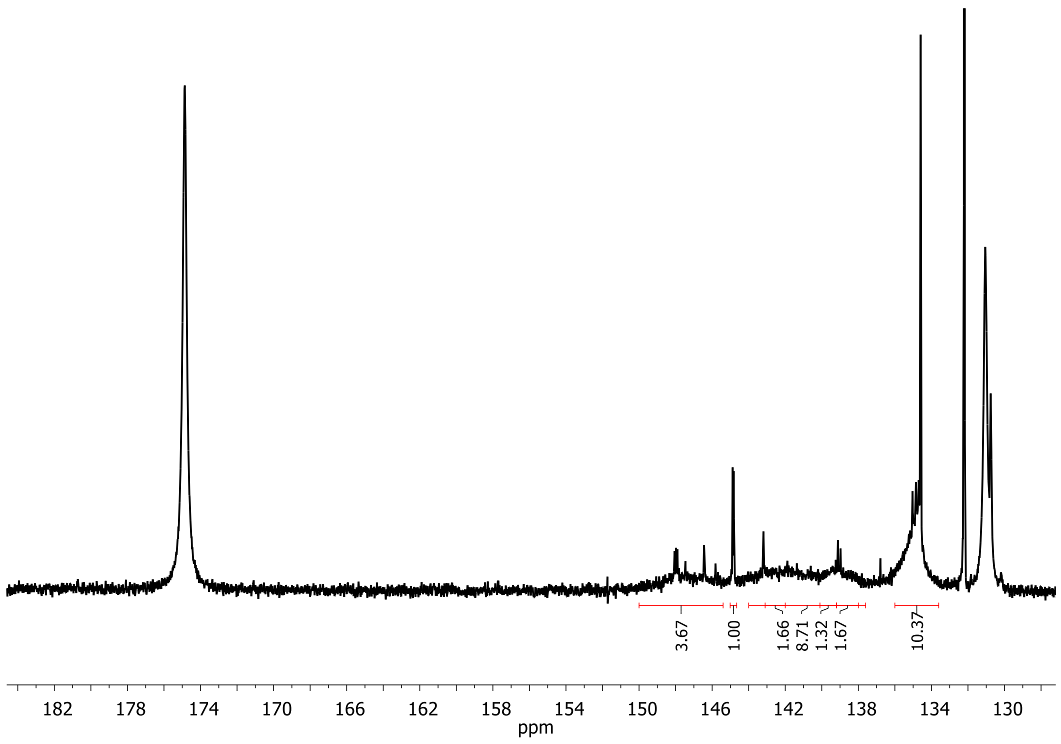

Figure S19. ^{31}P NMR spectrum of LB-pH1-60°C-×25.

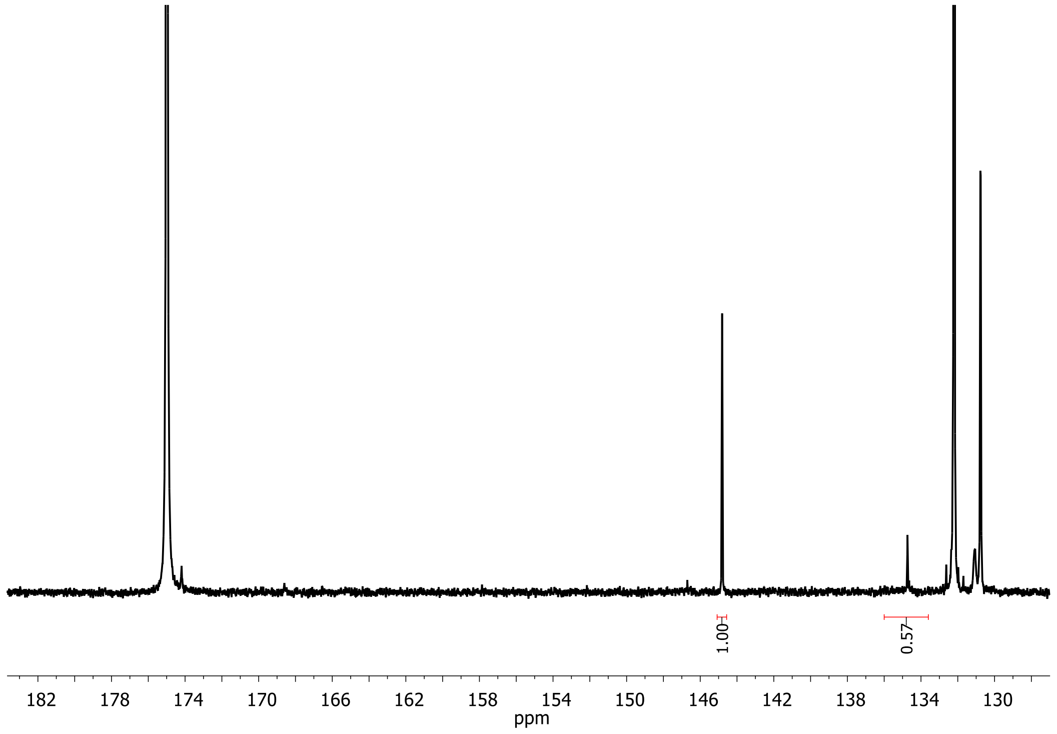

Figure S20. ^{31}P NMR spectrum of PolyActDLB.

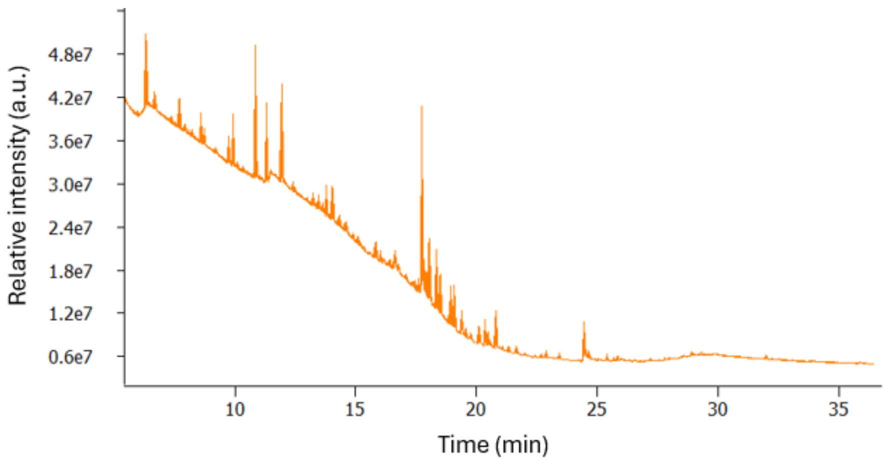

Figure S21. GC-FID-(TOF-MS) chromatogram of LB-pH1-50°C (7 h).

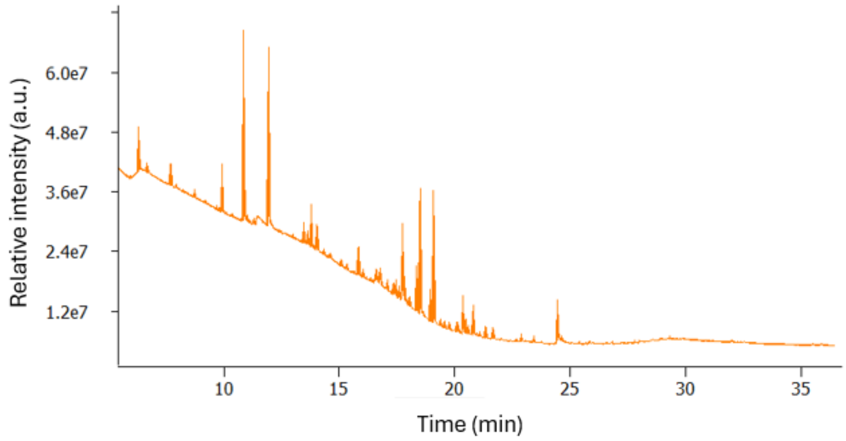

Figure S22. GC-FID-(TOF-MS) chromatogram of LB-pH1-60°C (3 h).

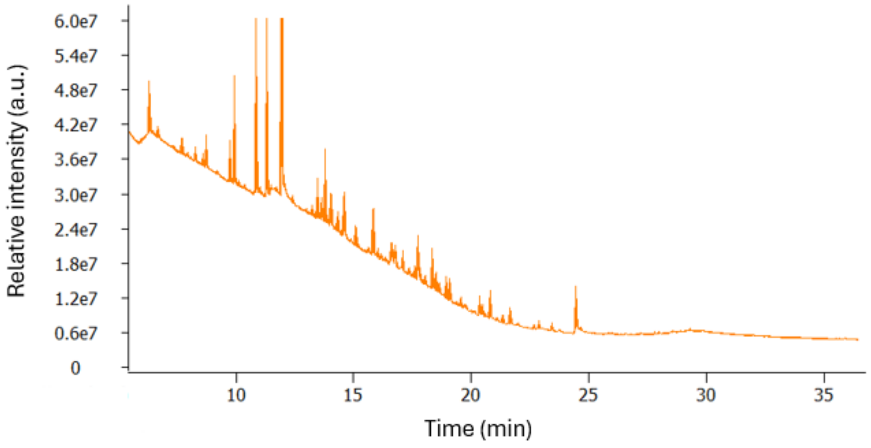

Figure S23. GC-FID-(TOF-MS) chromatogram of LB-pH1-70°C (2 h).

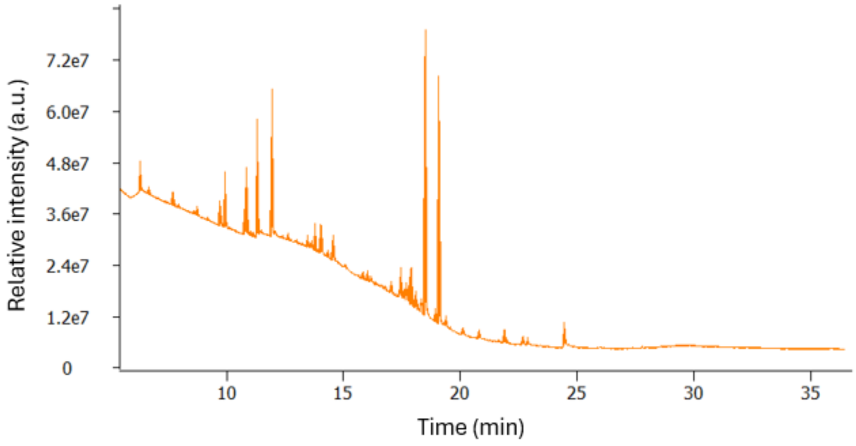

Figure S24. GC-FID-(TOF-MS) chromatogram of KL-pH1-60°C (3 h).

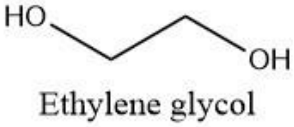

Figure S25. Molecular structures of the alcohols identified in the depolymerized lignin samples by GC-FID-(TOF-MS) analysis.

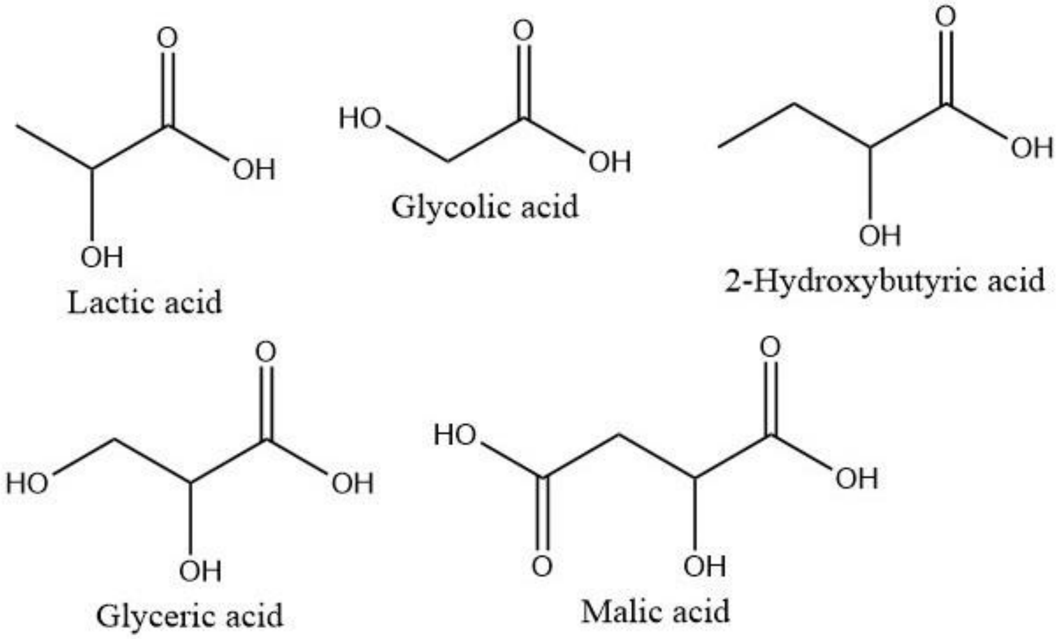

Figure S26. Molecular structures of the hydrocarboxylic acids identified in the depolymerized lignin samples by GC-FID-(TOF-MS) analysis.

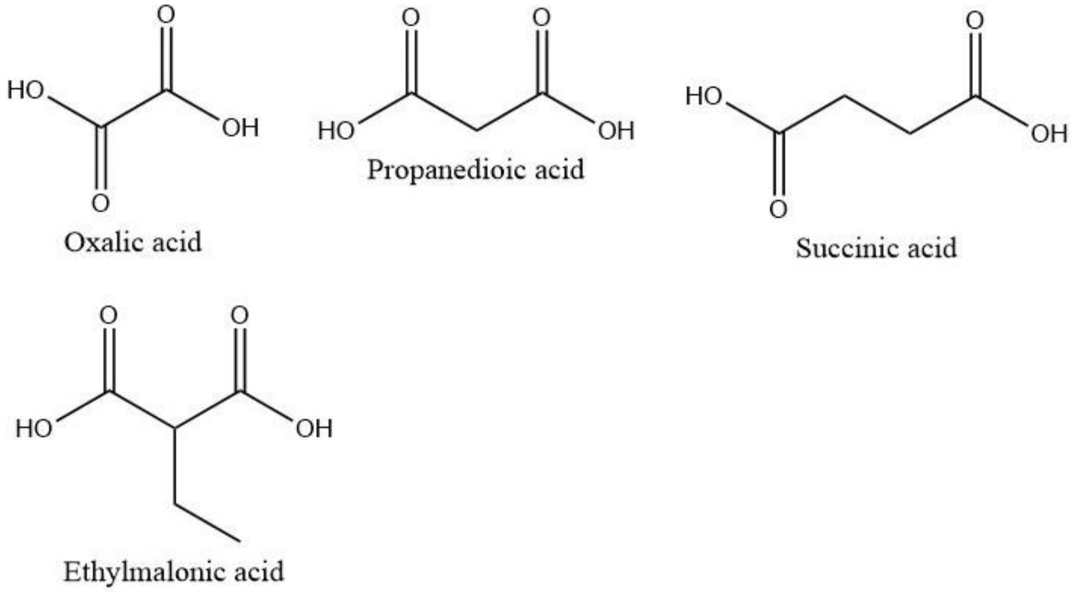

Figure S27. Molecular structures of the dicarboxylic acids identified in the depolymerized lignin samples by GC-FID-(TOF-MS) analysis.

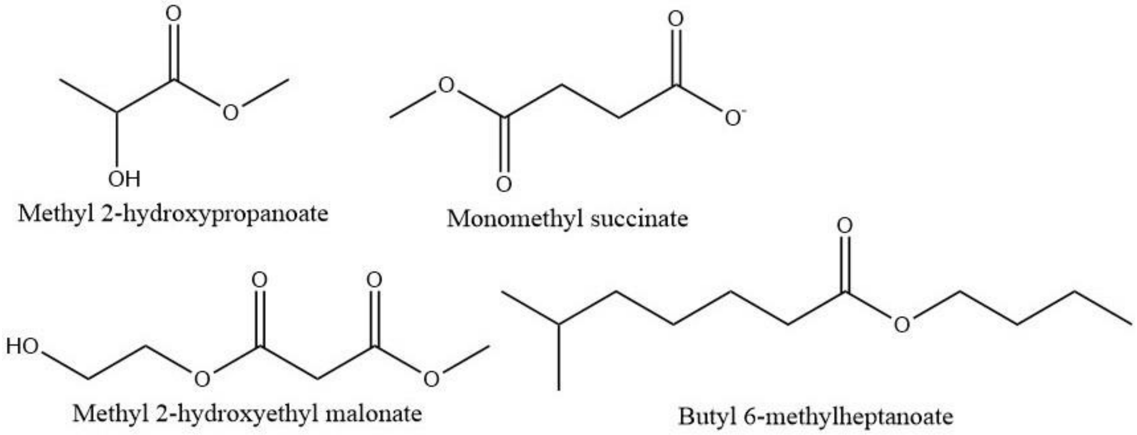

Figure S28. Molecular structures of the esters identified in the depolymerized lignin samples by GC-FID-(TOF-MS) analysis.

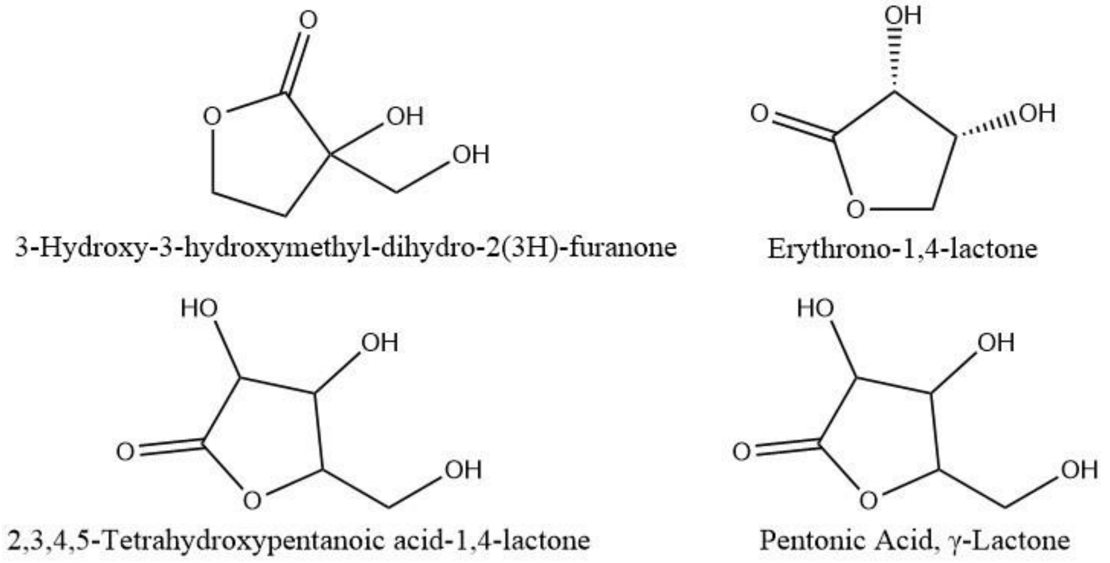

Figure S29. Molecular structures of the lactones identified in the depolymerized lignin samples by GC-FID-(TOF-MS) analysis.

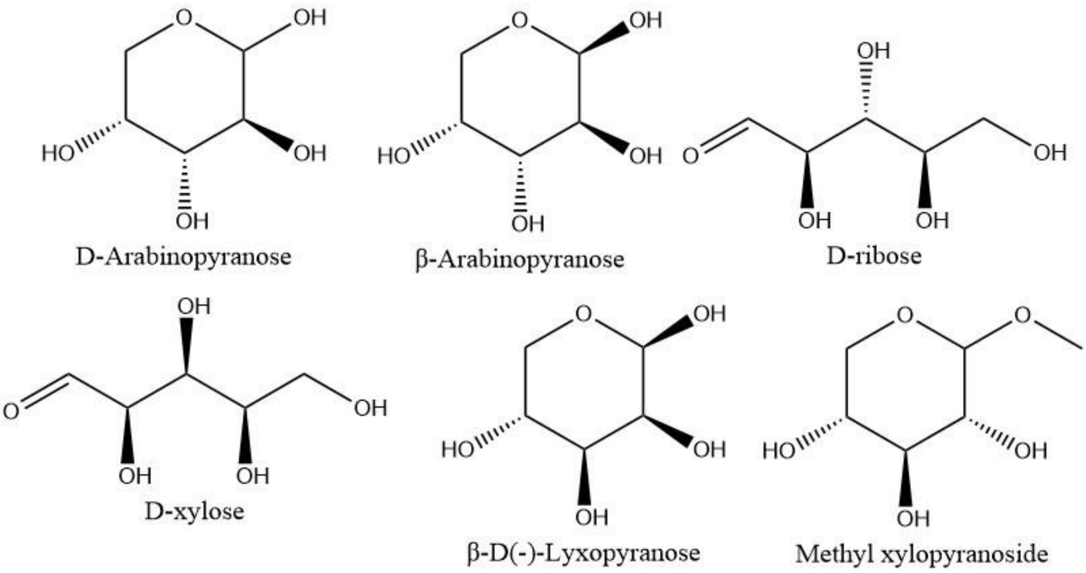

Figure S30. Molecular structures of the monosaccharides identified in the depolymerized lignin samples by GC-FID-(TOF-MS) analysis.

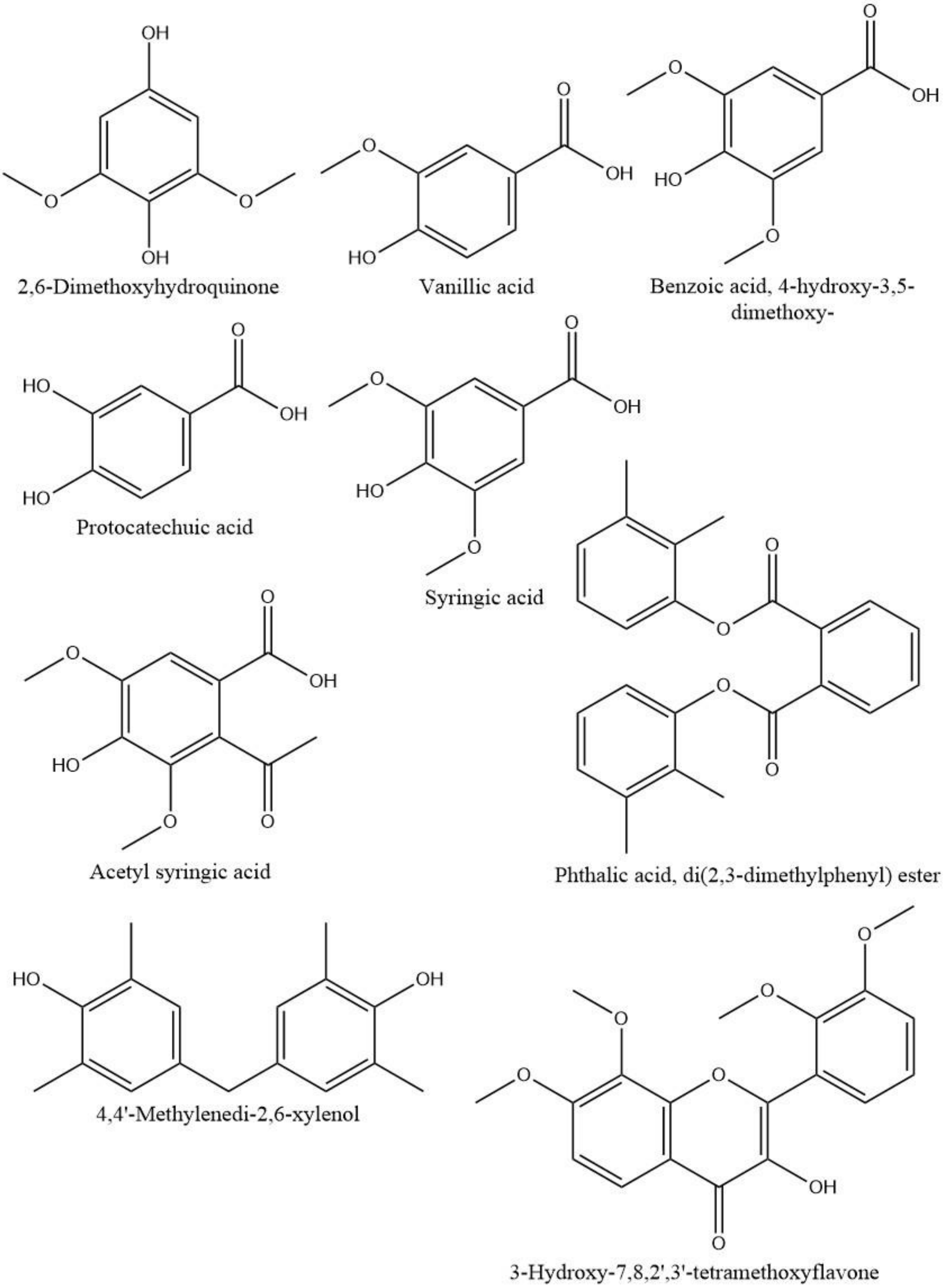

Figure S31. Molecular structures of the aromatics identified in the depolymerized lignin samples by GC-FID-(TOF-MS) analysis.
